# Effects of mifepristone on adipocyte differentiation in mouse 3T3-L1 cells

**DOI:** 10.1186/s11658-024-00559-9

**Published:** 2024-03-29

**Authors:** Takeshi Hashimoto, Katsuya Hirano

**Affiliations:** Department of Cardiovascular Physiology, Faculty of Medicine, Kagawa University, 1750-1 Miki-Cho, Kita-Gun, Kagawa, 761-0793 Japan

**Keywords:** Mifepristone, Adipocyte differentiation, PPARγ, siRNA, Neutralizing antibodies

## Abstract

**Background:**

Both glucocorticoid receptor and peroxisome proliferator-activated receptor-γ (PPARγ) play a critical role in adipocyte differentiation. Mifepristone is not only an antagonist of the glucocorticoid receptor but also an agonist of PPARγ. Therefore, the present study investigated the effect of mifepristone on adipocyte differentiation.

**Methods:**

Mouse 3T3-L1 cells were used as a model for adipocyte differentiation. The lipid droplet formation was evaluated with Bodipy493/503 staining and the expression of adipocyte markers [adiponectin and adipocyte fatty acid binding protein-4 (Fabp4)] was evaluated with quantitative PCR and immunoblot analyses for indication of adipocyte differentiation. siRNA and neutralizing antibodies were used to elucidate the molecular mechanism of mifepristone-induced adipocyte differentiation. Luciferase reporter assay was used to examine the effect of mifepristone on the promoter activity of PPAR-response element (PPRE). The DNA microarray analysis was used to characterize the transcriptome of the mifepristone-induced adipocytes. In vivo adipogenic effect of mifepristone was examined in mice.

**Results:**

Mifepristone not only enhanced adipocyte differentiation induced by the conventional protocol consisting of insulin, dexamethasone and 3-isobutyl-1-methylxanthine but also induced adipocyte differentiation alone, as evidenced by lipid droplets formation and induction of the expression of adiponectin and Fabp4. These effects were inhibited by an adiponectin-neutralizing antibody and a PPARγ antagonist. Mifepristone activated the promoter activity of PPRE in a manner sensitive to PPARγ antagonist. A principal component analysis (PCA) of DNA microarray data revealed that the mifepristone-induced adipocytes represent some characteristics of the in situ adipocytes in normal adipose tissues to a greater extent than those induced by the conventional protocol. Mifepristone administration induced an increase in the weight of epididymal, perirenal and gluteofemoral adipose tissues.

**Conclusions:**

Mifepristone alone is capable of inducing adipocyte differentiation in 3T3-L1 cells and adipogenesis in vivo. PPARγ plays a critical role in the mifepristone-induced adipocyte differentiation. Mifepristone-induced adipocytes are closer to the in situ adipocytes than those induced by the conventional protocol. The present study proposes a single treatment with mifepristone as a novel protocol to induce more physiologically relevant adipocytes in 3T3-L1 cells than the conventional protocol.

**Supplementary Information:**

The online version contains supplementary material available at 10.1186/s11658-024-00559-9.

## Background

World Health Statistics 2023 reported that the worldwide prevalence of obesity among adults of ≥ 18 years of age was 13.1%, affecting approximately 650 million people, in 2016 (World Health Organization, Geneva, Switzerland). Obesity causes a global health burden as one of the major risk factors for noncommunicable diseases, such as cardiovascular diseases, diabetes and some types of cancer. A better understanding of the underlying mechanisms is fundamental for overcoming this public health issue. Obesity is characterized by increased adipose tissue mass, which can be attributed to either an increase in adipocyte size (hypertrophy) or an increase in adipocyte number (hyperplasia) due to adipogenesis from adipocyte precursors [1]. Hypertrophy is more relevant to metabolically unhealthy pathological obesity, whereas hyperplasia is more relevant to metabolically healthy physiological obesity [1]. Adipocytes are derived from mesenchymal stem cells through two stages of differentiation: commitment of mesenchymal stem cells to preadipocytes and terminal differentiation of preadipocytes to mature adipocytes [2]. Both stages are regulated by the well-orchestrated action of multiple extracellular signals, corresponding intracellular signals and sequential activation of multiple transcription factors [1–6]. Among the transcription factors, CCAAT/enhancer-binding proteins (C/EBPs) and peroxisome proliferator-activated receptor-γ (PPARγ) play central roles in the terminal differentiation of adipocytes [1–6]. C/EBPβ and C/EBPδ are rapidly induced by adipocyte differentiation factors and contribute to the early phase of terminal differentiation and subsequent induction of PPARγ [2, 4]. PPARγ, in association with C/EBPα, contributes to the later phase of terminal differentiation and maintenance of the mature adipocyte phenotype by regulating the expression of adipocyte marker proteins [2–4]. Adiponectin, leptin, adipocyte triglyceride lipase, lipoprotein lipase and perilipin 1 are representative mature adipocyte markers [1]. The adiponectin gene is one of the target genes regulated by PPARγ [7].

A cell culture model of adipocyte differentiation has contributed significantly to elucidating the molecular mechanisms of terminal differentiation [6]. Among the various preadipocytes and extracellular factors reported [6], 3T3-L1 cells and a combination of isobutyl-methylxanthine (IBMX), dexamethasone and insulin are widely used as representative models and in the conventional protocol of adipocyte differentiation [6, 8]. Cyclic AMP (cAMP) plays an important role in adipocyte differentiation, mainly by phosphorylating and activating the transcription factor cAMP-response element binding protein (CREB), which in turn activates the transcription of the C/EBPβ gene [2, 4, 5]. In the conventional protocol of adipocyte differentiation, isobutyl-methylxanthine, an inhibitor of cAMP phosphodiesterase activity, is considered to increase cellular cAMP levels and activate the CREB-C/EBPβ pathway, thereby promoting adipocyte differentiation. Dexamethasone induces the expression of C/EBPα, activates the transcriptional activity of C/EBPβ by increasing its acetylation, and induces the expression of C/EBPδ—all partly via glucocorticoid receptors—thereby contributing to adipocyte differentiation [1, 2]. Insulin induces the expression of PPARγ via a pathway involving Akt and the mammalian target of rapamycin through either the canonical insulin receptor or insulin-like growth factor receptor, thereby contributing to adipocyte differentiation [1, 3].

Mifepristone is an antagonist of the progesterone and glucocorticoid receptor [9–12] and an anti-conceptive drug approved by the Food and Drug Administration of the United States of America. Mifepristone has been reported to directly bind to PPARγ and act as a partial agonist of PPARγ [13, 14]. We previously reported that mifepristone prevents high-fat diet-induced insulin resistance, hepatic steatosis, and the downregulation of adiponectin [14]. Notably, mifepristone prevented adipocyte hypertrophy induced by a high-fat diet. In 3T3-L1 cells, mifepristone enhanced the expression of adiponectin in a manner that was sensitive to PPARγ inhibition and siRNA-mediated knockdown of PPARγ. It is thus suggested that mifepristone prevents pathological obesity due to adipocyte hypertrophy, probably owing to its enhancement of the expression and secretion of adiponectin, which is attributed to the improvement of insulin resistance induced by a high-fat diet. However, the effects of mifepristone on adipocyte differentiation and adipogenesis remain unclear. The antagonistic effect on the glucocorticoid receptor may counteract the agonistic effect of PPARγ during adipocyte differentiation.

The present study aimed to elucidate the effect of mifepristone on adipocyte differentiation in 3T3-L1 cells, with and without the conventional protocol of adipocyte differentiation. The effect of mifepristone on in vivo adipogenesis was also investigated in mice fed a regular diet. The present study demonstrates that mifepristone itself induces adipocyte differentiation probably through activation of PPARγ transcriptional activity and the induction of adiponectin, although some factors in serum added to the culture media are required for full differentiation. A transcriptome analysis revealed that mifepristone-induced adipocytes were closer to in situ adipocytes in normal adipose tissues than those induced by the conventional protocol. The present study proposes a single treatment with mifepristone as a new protocol for studying adipocyte differentiation using 3T3-L1 cells. This protocol may be useful for investigating adipocyte differentiation under physiological conditions.

## Materials and methods

### Reagents

Mifepristone (a glucocorticoid receptor antagonist) and T0070907 (a peroxisome proliferator-activated receptor-γ [PPARγ] antagonist) were purchased from Cayman Chemical (Ann Arbor, MI, USA). Primers for real-time quantitative RT-PCR (qRT-PCR) were synthesized by Eurofins Genomics Inc. (Tokyo, Japan). Fetal bovine serum (FBS) was purchased from JRH Biosciences (Lenexa, KS, USA). The neutralizing antibody for adiponectin (NAb, ANOC 9104) was a kind gift from Prof. Iichirou Shimomura (Osaka University Graduate School of Medicine, Japan). Other reagents were purchased from Sigma (St. Louis, MO, USA) unless otherwise noted.

### Cell culture of 3T3-L1 cells, adipocyte differentiation and treatment with mifepristone

**Fig. 1:**
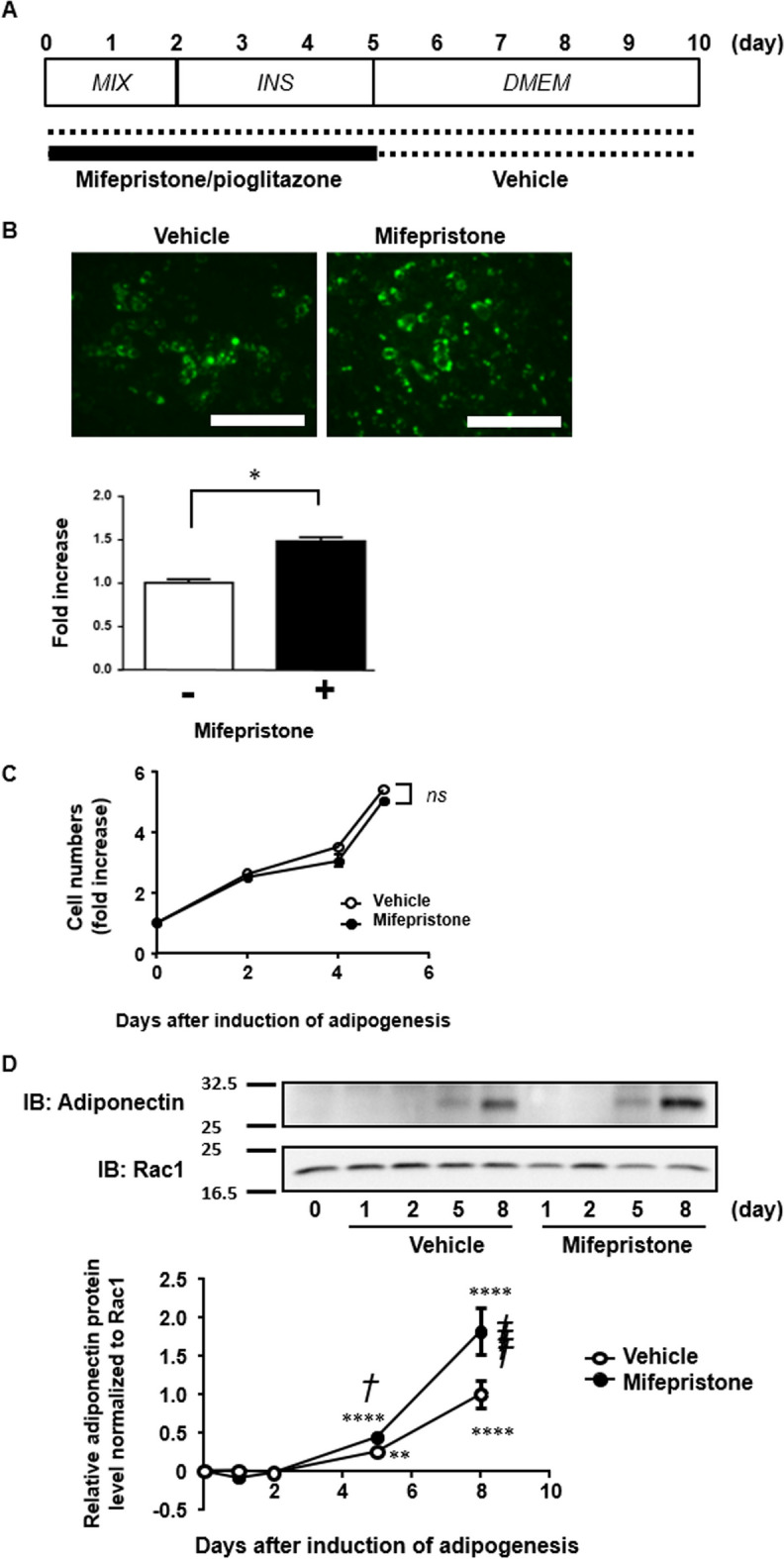

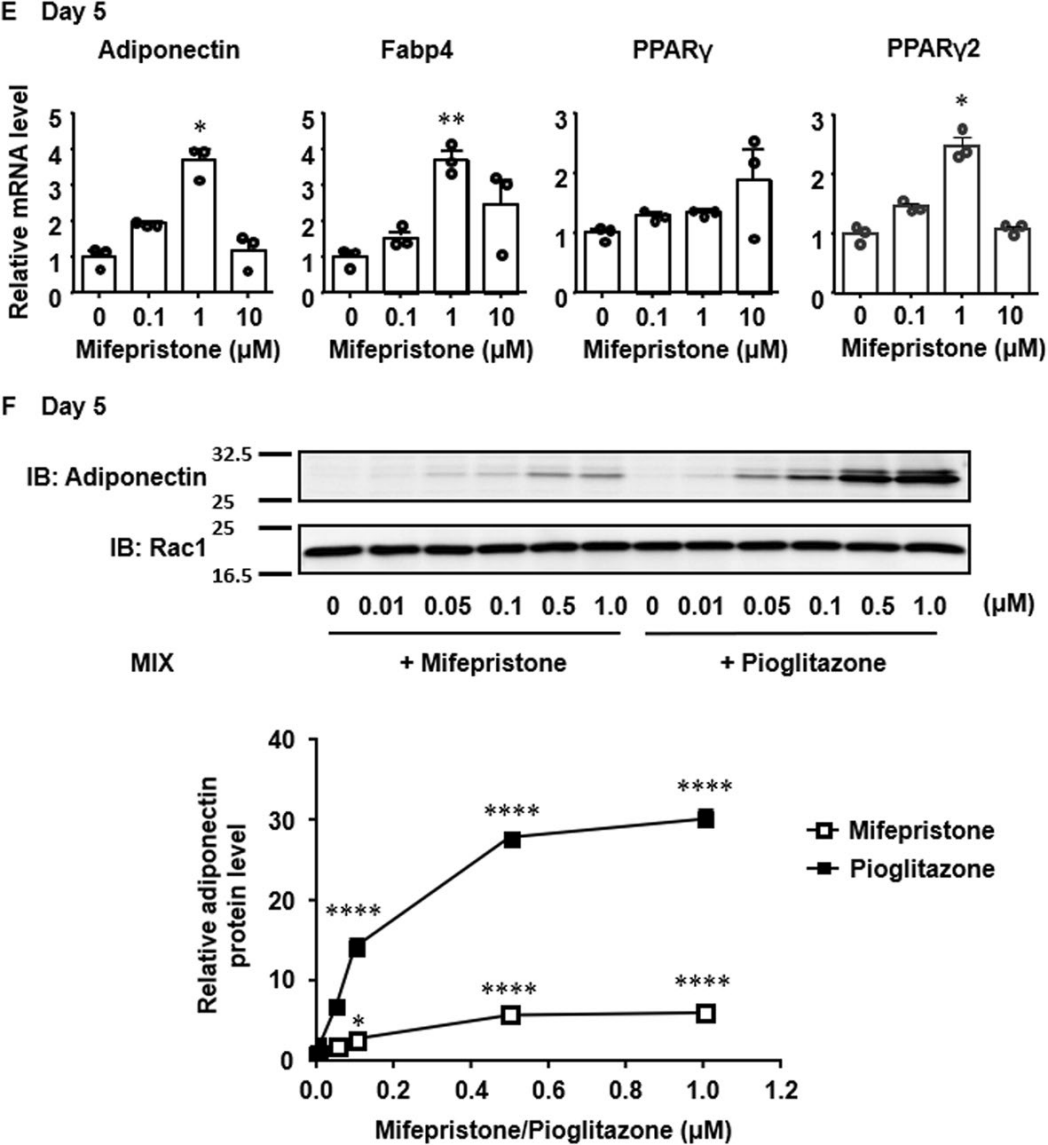
Augmentation by mifepristone of upregulation of adiponectin expression during adipocyte differentiation in 3T3-L1 cells. **A** The experimental protocol used to examine the effect of treatment with vehicle (dotted line) and either mifepristone or pioglitazone (solid line) during the differentiation of 3T3-L1 cells to adipocytes. Adipocyte differentiation was induced as described in the Materials and Methods. MIX, the period of incubation with the first differentiation medium, containing 4.5 g/L D-glucose, 1 µg/mL insulin, 1 µM dexamethasone and 0.5 mM IBMX in the standard culture media. INS, the period of incubation with the second differentiation medium, consisting of 4.5 g/L D-glucose and 1 µg/mL insulin in the standard culture media. DMEM, the period of incubation with the standard culture media. **B** Representative microscopic images of Bodipy staining and summaries (*n =* 6) of the quantification on day 10 after induction of adipogenesis. Scale bar, 600 µm. **C** The time-dependent changes in the cell count during adipogenesis with and without 1 µM mifepristone. **D** Representative immunoblot images and summary (*n =* 4) showing the time course of changes in the adiponectin and Rac1 protein expression during adipogenesis with and without treatment with 1 µM mifepristone. The levels of adiponectin were normalized by those of Rac1, and the value obtained without mifepristone treatment on day 8 was assigned a value of 1. **E** Summary (*n =* 6) of the qRT-PCR analysis of the expression of adiponectin, Fabp4, PPARγ and PPARγ2 mRNA on day 5. The level of each transcript was normalized to that of 18S rRNA, and the value obtained without treatment was assigned a value of 1. **F** Representative Immunoblot (IB) images and summary (*n =* 4) of the concentration-dependent effects of treatment with mifepristone or pioglitazone on the adiponectin protein expression on day 5. The expression levels of adiponectin were normalized to those of Rac1, and then expressed as the fold increase from the value obtained without treatment. All data represent the mean ± S.E.M. *, *P* < 0.05; **, *P* < 0.01; ***, *P* < 0.001; ****, *P* < 0*.*0001 vs. Day 0 (**B**) or the values obtained without treatment (**C**, **D**). †*P* < 0*.*05; ††††, *P* < 0*.*0001 vs. vehicle. ns, not significantly different

**Fig. 2:**
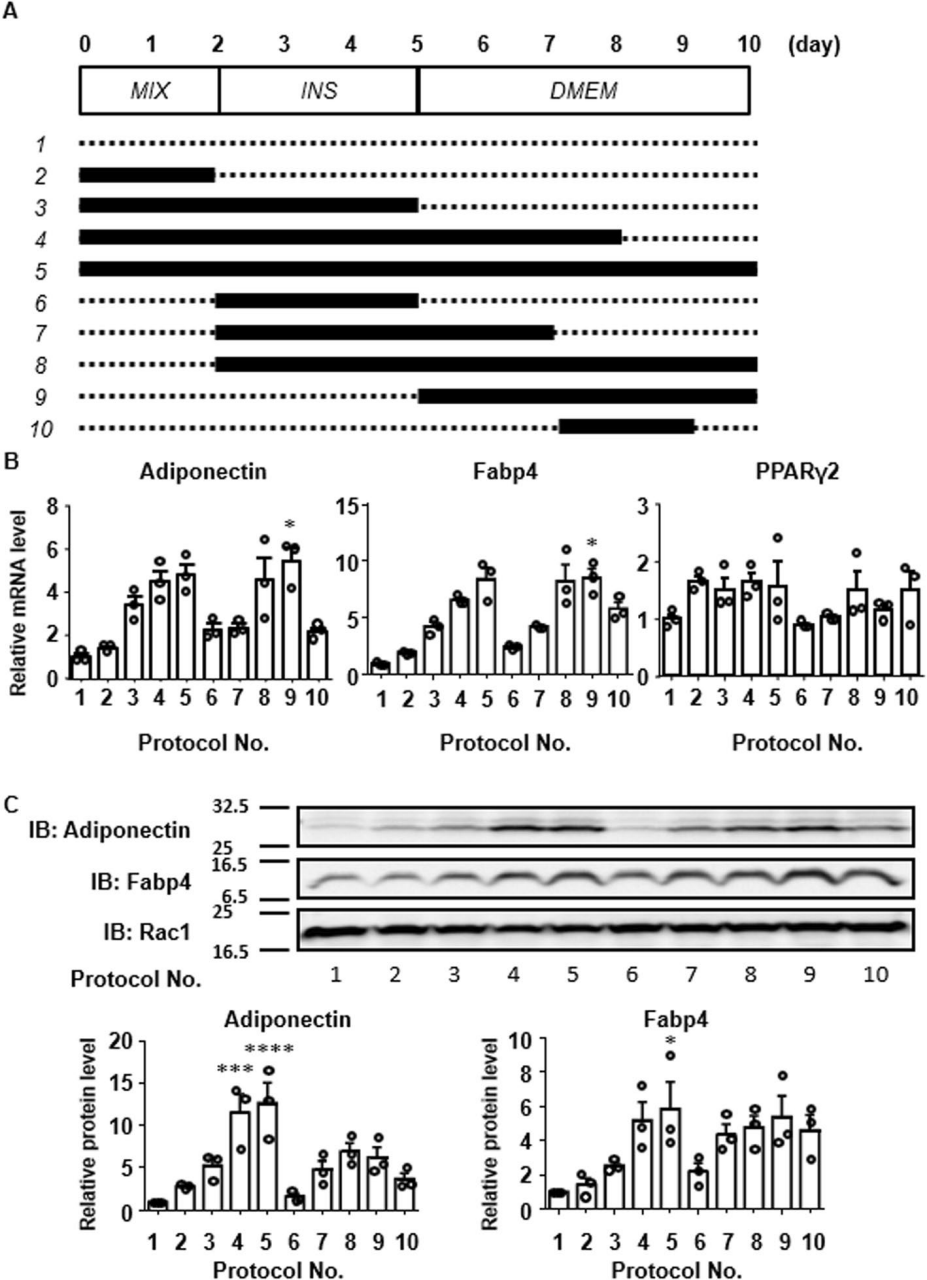
Critical period of mifepristone treatment for augmenting the adiponectin expression during adipocyte differentiation in 3T3-L1 cells. **A** Experimental protocols used to determine the critical period of treatment with 1 µM mifepristone (solid lines) for augmenting the expression of adiponectin during adipogenesis. Confluent 3T3-L1 cells were subjected to adipocyte differentiation as described in the Materials and Methods. The dotted lines indicate the period of vehicle treatment. **B** Summary (*n =* 6) of the qRT-PCR analysis of the expression of adiponectin, Fabp4, and PPARγ2 mRNA on day 10. The level of each transcript was normalized to that of 18S rRNA, and the value obtained with protocol 1 was assigned a value of 1. **C** Representative immunoblot (IB) images and summary (*n =* 4) of the expression of adiponectin and Fabp4 proteins on day 10. The levels of adiponectin and the Fabp4 expression were normalized by those of Rac1, and then expressed as the fold increase from the values obtained with protocol 1. All data represent the mean ± S.E.M. *, *P* < 0.05; **, *P* < 0.01 vs. values obtained with protocol 1 (vehicle alone)

**Fig. 3:**
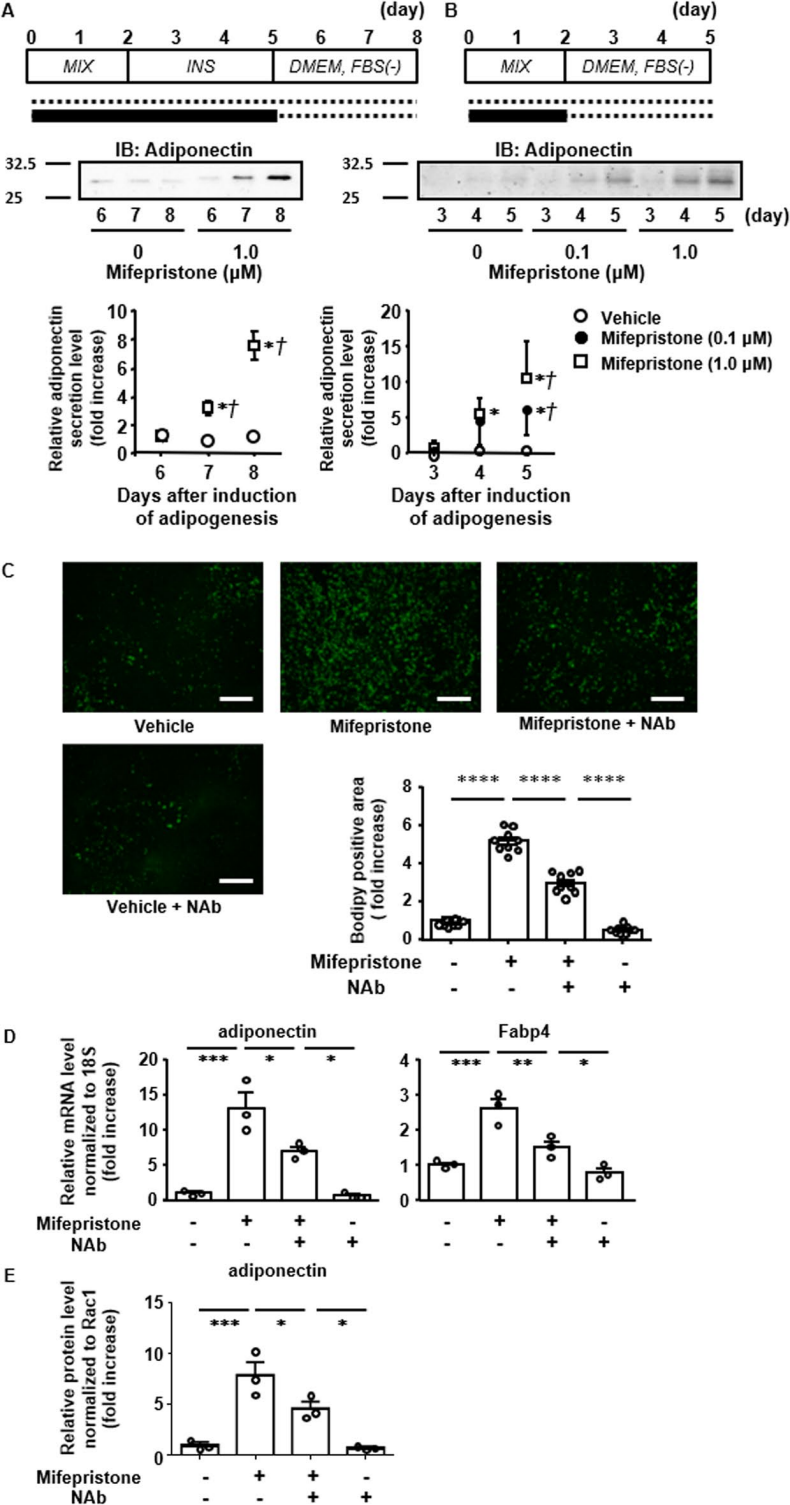

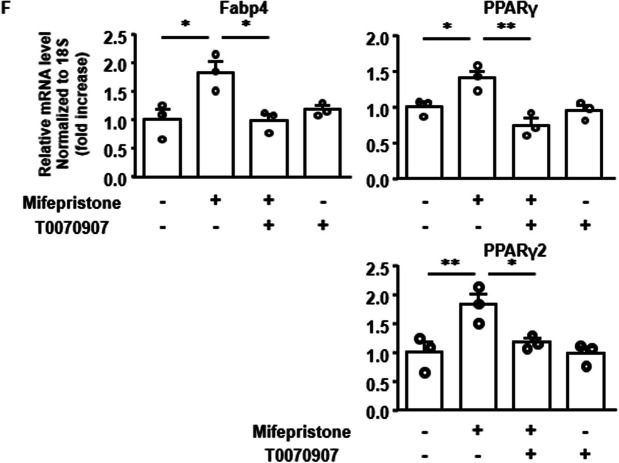
Evaluation of the paracrine/autocrine effect of adiponectin in the mifepristone-induced enhancement of adipocyte differentiation in 3T3-L1 cells. **A**, **B** The effect of mifepristone on the secretion of adiponectin during adipogenesis was examined in two protocols. Representative immunoblot (IB) images and summary (*n =* 4) show the levels of adiponectin secretion with and without mifepristone treatment (0.1 and 1 µM) on days 6, 7 and 8 (**A**) and days 3, 4 and 5 (**B**). The adiponectin secretion levels were expressed relative to those obtained without mifepristone on day 6 (**A**) or day 3 (**B**). In the schematic illustrations showing the experimental protocols, the dotted lines indicate the period of vehicle treatment, while the solid lines indicate the period of mifepristone treatment. Since bovine adiponectin in FBS interferes with the quantification of secreted mouse adiponectin, the samples were obtained in serum-free medium. **C** Representative fluorescent microscopic images and summary (*n =* 4) of Bodipy 493/503 (green) fluorescence on day 12 after the induction of adipogenesis, with and without treatment with 1 µM mifepristone and 12.5 µg/mL adiponectin-neutralizing antibody ANOC 9104 (NAb). Mifepristone and NAb were applied during 5-day treatment with MIX and INS medium. Scale bar, 300 µm. **D**, **E** The effects of mifepristone and NAb on the expression of adiponectin and Fabp4 mRNA (**D**) and adiponectin protein (**E**) on day 5 after the induction of adipogenesis. The mRNA level was normalized to that of 18S rRNA; the protein level was normalized to that of Rac1. The values obtained with no treatment were assigned a value of 1. **F** Summary (*n =* 3) of the effect of 1 µM mifepristone and 10 µM T0070907 on the expression of Fabp4, PPARγ, and PPARγ2 mRNA on day 5 after the induction of adipogenesis according to the protocol shown in Fig. 1A. The levels of Fabp4 and PPARγ were normalized to the level of 18S rRNA, and expressed relative to that obtained without any treatment. All data represent the mean ± S*.*E*.*M. *P < 0*.*05; **P < 0*.*01 vs. day 6 (**A**), day 3 (**B**) or as indicated (**C**–**F**). ^†^*P* < 0*.*05; ^††^*P* < 0.01 vs. vehicle (**A** and **B**)

**Fig. 4:**
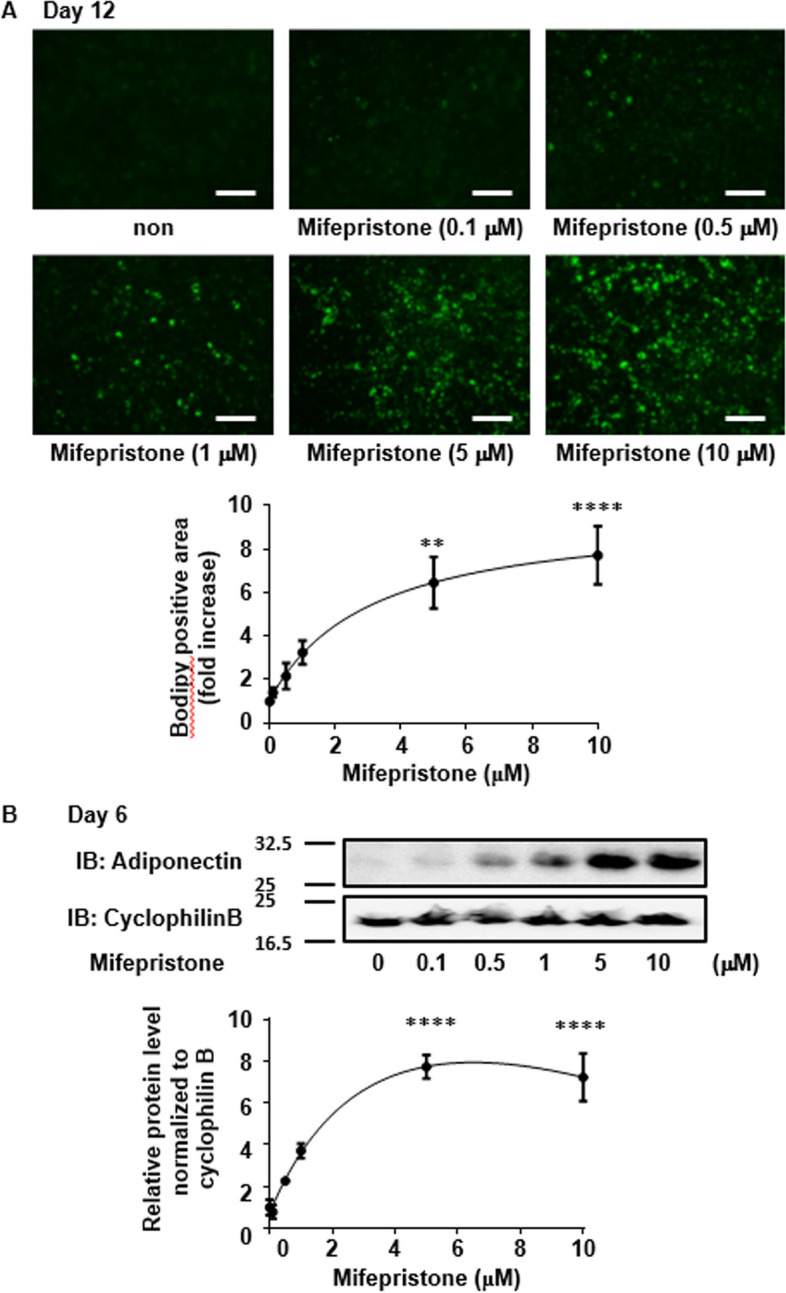
Induction of adipocyte differentiation and the expression of adiponectin by mifepristone alone in 3T3-L1 cells. **A** Representative fluorescent microscopic images of Bodipy 493/503 (green) fluorescence and summary (*n =* 3) of the concentration-dependent effects of mifepristone on the lipid accumulation on day 12. The cells were treated with mifepristone for the first 5 days, and then cultured in its absence until day 12. Scale bar, 300 µm. **B** Representative immunoblot (IB) images and summary (*n =* 3) of the concentration-dependent effects of treatment with mifepristone on the adiponectin protein expression on day 6. The expression levels of adiponectin were normalized to those of cyclophilin B, and then expressed as the fold increase from the value obtained without treatment. All data represent the mean ± S.E.M. *P < 0.05; **P < 0.01; ***P < 0.001; ****P < 0*.*0001 vs. the values obtained without treatment

**Fig. 5:**
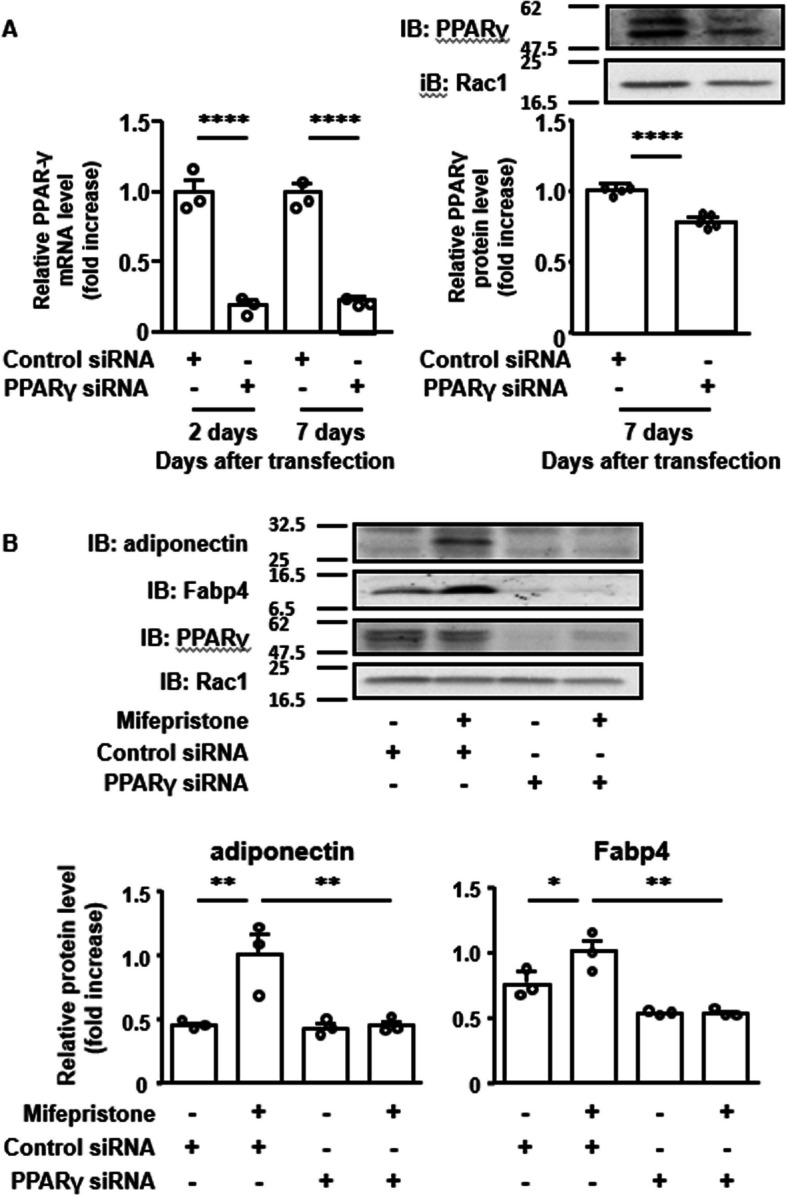
Effects of knockdown of PPARγ on the expression of adiponectin and Fabp4 during mifepristone-induced adipocyte differentiation in 3T3-L1 cells. **A** Summary of the qRT-PCR (*n =* 3) and immunoblot (IB) (*n =* 4) analyses of the efficacy of knockdown of PPARγ on 2 and 7 days after transfection of siRNA (10 nM). Representative IB images are also shown. The levels of PPARγ mRNA and protein were normalized by those of 18S rRNA and Rac1, respectively, and the values obtained with control siRNA were assigned a value of 1. **B** Representative immunoblot (IB) images and summary (*n =* 3) of the effects of PPARγ knockdown on the mifepristone-induced expression of adiponectin and Fabp4 proteins on day 5 after the induction of adipogenesis (i.e., 7 days after transfection of PPARγ or control siRNA). The levels of adiponectin and the Fabp4 protein expression were normalized to those of Rac1, and the values obtained with mifepristone and control siRNA were assigned a value of 1. All data represent the mean ± S.E.M. *P < 0*.*05; **P < 0*.*01

**Fig. 6:**
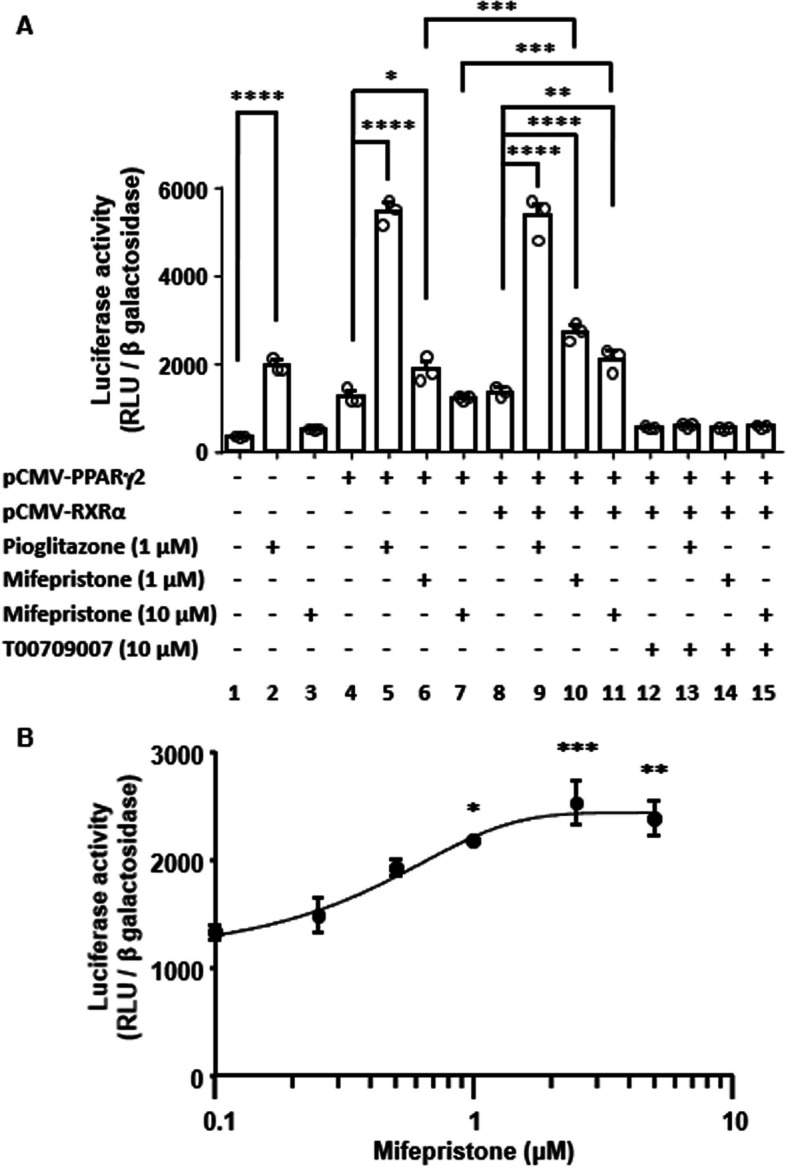
Effect of mifepristone on the activity of promoter containing PPAR response elements and its dependence on the co-expression of PPARγ2 and RXRα in COS7 cells. **A** The concentration-dependent effects of mifepristone on the activity of promoter containing three copies of PPAR response element in COS7 cells, which were co-transfected with PPARγ2 and RXRα expression vectors. **B** Summary of the effects of mifepristone (1 and 10 µM) and pioglitazone (1 µM) on the promoter activity of PPAR response element with and without the co-expression of PPARγ2 and RXRα or co-treatment with the PPARγ2 antagonist, T00709007 (10 µM), as indicated. The data represent the mean ± S.E.M. of triplicate measurements of one set of experiments. Similar results were obtained with an additional two sets of experiments

Mouse 3T3-L1 cells (JCRB9014; JCRB Cell Bank, Osaka, Japan) were cultured in non-coated cell cultured dishes and differentiated into adipocytes using a previously reported, conventional protocol [15]. Briefly, 3T3-L1 cells were maintained in Dulbecco’s modified Eagle’s medium (DMEM) containing 1.0 g/L D-glucose, 10% FBS, 100 U/mL penicillin, 0.1 mg/mL streptomycin, 4 mM l-glutamine, and 3.7 g/L NaHCO_3_, at 37 °C in a 5% CO_2_ incubator. As depicted in Fig. 1A, adipocyte differentiation was induced 2 days after reaching confluence (defined as day 0) by switching the standard medium (DMEM) to the first differentiation medium (MIX), which contained 4.5 g/L D-glucose, 1 µg/mL insulin (INS), 1 µM dexamethasone and 0.5 mM 3-isobutyl-1-methylxanthine (IBMX). Two days later, the MIX medium was changed to the second differentiation medium (INS), which was equivalent to MIX, except for the absence of dexamethasone and IBMX. Three days later, INS medium was replaced with the standard DMEM medium. The cells were then further incubated for full differentiation, and the medium was renewed every second day. Cells were treated either with mifepristone or vehicle (ethanol) according to the protocol indicated in Figs. 1A, 2A, 3A and B. Cell numbers were determined using a particle counter (CDA-1000B, SYSMEX, Kobe, Japan). The single treatment with mifepristone (Figs. 4, 5, and 6) was conducted in DMEM containing 4.5 g/L D-glucose, 10% FBS, 100 U/mL penicillin, 0.1 mg/mL streptomycin, 4 mM l-glutamine, and 3.7 g/L NaHCO_3_, at 37 °C in a 5% CO_2_ incubator.

### Quantification of lipid accumulation with Bodipy 493/503 staining

The cells were washed with PBS and fixed with 4% (w/v) paraformaldehyde in PBS (Wako Pure Chemical Industries, Ltd., Osaka, Japan) for 30 min. They were then co-stained with a solution comprising 3 µg/mL Bodipy 493/503 and 7.5 µg/mL 4′,6-diamidino-2-phenylindole (DAPI) in water for 20 min at room temperature and then washed twice with PBS. Fluorescence images were captured using a BioRevo BZ9000 fluorescence microscope (Keyence, Osaka, Japan) and saved as TIFF files. The Bodipy 493/503- and DAPI-positive areas were quantified using a Hybrid Cell Count (Keyence, Osaka, Japan). The Bodipy 493/503-positive area was normalized to that of DAPI-positive area to estimate the number of cells recruited to adipocyte differentiation.

### Immunoblot analyses of total cell lysates

Total cellular proteins were extracted using Cell Lysis Buffer (cat.9803; Cell Signaling Technology, Beverly, MA, USA). The protein concentration was determined using a Bicinchoninate Protein Assay Kit (Nacalai, Kyoto, Japan) with bovine serum albumin as a standard. Protein samples were subjected to gel electrophoresis and transferred to membranes as previously described [15]. The cells were blocked with Super Block blocking buffer (Thermo Fisher Scientific, Rockford, IL, USA). The membranes were probed with primary antibodies raised against 30 kDa for adiponectin (A6354; Sigma), 15 kDa for adipocyte fatty acid binding protein-4 (Fabp4) (cat.3544; Cell Signaling Technology), 53 and 57 kDa for PPARγ (cat.2430; Cell Signaling Technology), 21 kDa for Rac1 (cat.BD610650; BD Biosciences, Franklin Lakes, NJ, USA) and 21 kDa for cyclophilin B (cat.ab16045; Abcam, Cambridge, MA, USA), followed by incubation with the corresponding secondary antibodies conjugated with horseradish peroxidase (HRP). The immune complex was visualized using an enhanced chemiluminescence reagent, SuperSignal West Pico Chemiluminescent Substrate (Thermo Fisher Scientific). The chemiluminescence signal was detected using a LAS1000-Plus chemiluminescence image analyzer (Fujifilm Co, Tokyo, Japan) and quantified using the Image Studio software program (version 4.0; LI-COR).

### Immunoblot analysis of adiponectin in culture medium

Adiponectin secretion into the culture medium of 3T3-L1 cells was quantified by an immunoblot analysis, as previously reported [15]. Cells were cultured in serum-free medium for 3 days before collecting the culture medium, because bovine adiponectin in FBS interferes with the immunodetection of mouse adiponectin. The same amount of culture medium was subjected to an immunoblot analysis to evaluate the amount of adiponectin secreted in the medium. The collected culture medium was mixed with an equal volume of 2 × Laemmli’s sample buffer. The immunoblot analysis of adiponectin levels was performed as described above.

### RNA preparation and qRT-PCR

Total RNA was isolated from 3T3-L1 cells and reverse-transcribed into cDNA as previously reported [14]. The resulting template cDNA was subjected to real-time PCR according to the manufacturer’s instructions (Roche Diagnostics, Basel, Switzerland). Briefly, real time PCR was performed using TaqMan Universal PCR Master Mix and Universal Probe Library probe. Fluorescence-labelled TaqMan MGB probes (200 nM) were used for data collection during the log-linear phase of the reaction. PCR primers for mouse adiponectin, leptin, PPARγ, Fabp4, and 18S rRNA were designed using the ProbeFinder program at the Assay Design Center of Universal ProbeLibrary (Roche Diagnostics) (https://qpcr.probefinder.com/organism.jsp) and synthesized by Eurofins Genomics Japan (Kyoto, Japan). The sequences of the PCR primers were as follows: adiponectin, forward primer: 5′-GGA GAG AAA GGA GAT GCA GGT-3′ and reverse primer: 5′-CTT TCC TGC CAG GGG TTC-3′; PPARγ, forward primer: 5′-TTA TAG CTG TCA TTA TTC TCA GTG GAG-3′ and reverse primer: 5′-GAC TCT GGG TGA TTC AGC TTG-3′; Fabp4, forward primer: 5′-TCG ACC ACA ATA AAG AGA AAA CG-3′ and reverse primer: 5′-CTT GTG GAA GTC ACG CCT TT-3′; 18S rRNA, forward primer: 5′-CTC AAC ACG GGA AAC CTC AC-3′ and reverse primer: 5′-CGC TCC ACC AAC TAA GAA CG-3′. The TaqMan probes used for adiponectin, PPARγ, Fabp4, and 18S rRNA were Universal ProbeLibrary Probes No. 17, 62, 77, and 77, respectively. The expression of PPARγ2 mRNA was evaluated using real-time multiplex PCR with SYBR green (Takara Bio, Otsu, Japan). PCR primers for mouse PPARγ2 were designed using the Primer-BLAST (National Institute of Health, Bethesda, MD, USA) and synthesized by Eurofins Genomics Japan. The sequences of the PCR primers for mouse PPARγ2, transcript variant 2 (Accession No. NM_011146.4) are 5′-CTC CTG TTG ACC CAG AGC AT-3′ (forward primer) and 5′-CTT CCA TCA CGG AGA GGT CC-3′ (reverse primer). The PCR protocol consisted of an initial step of 2-min activation at 50 °C and 10-min denaturation at 95 °C, followed by 40 cycles of 15-s denaturation at 95 °C and 1-min extension at 60 °C. The Ct values of the target genes were analyzed using the QuantStudio™ Real-Time PCR software program from Thermo Fisher Scientific and performed calculations using the standard curves obtained with the known amount of the cDNA for each target. The mRNA levels of adiponectin, Fabp4, PPARγ, and PPARγ2 were expressed relative to those obtained for the corresponding 18S rRNA.

### siRNA-mediated knock down of PPARγ

Small interfering RNA (siRNA) specific for mouse PPARγ (5’-CUA CGA CAU GAA UUC CUU AAU-3’) [16] and MISSION^®^ siRNA Universal Negative Control siRNA were purchased from Sigma Genosys siRNA Service (Sigma). Two days before treatment with mifepristone alone, 3T3-L1 cells were transfected with siRNA by 5-h exposure to a transfection mixture containing 10 nM siRNA duplexes and 2.5 μL/mL Lipofectamine 2000 (Thermo Fisher Scientific). The cells were then treated with mifepristone at different concentrations for the period indicated in the figure legends.

### Luciferase reporter assay

The effect of mifepristone on the promoter activity of the PPAR response element (PPRE) was examined in COS7 cells (JCRB9127; JCRB Cell Bank, Osaka, Japan) using a reporter plasmid containing three copies of the PPRE and thymidine kinase promoter located upstream of a firefly luciferase gene (Addgene, catalog number1015; Cambridge, MA, USA). The expression plasmids for mouse PPARγ2 (pCMV-PPARγ2) and retinoid X receptor α (RXRα; pCMV-RXRα) were constructed in the pCMV-Myc vector (Clontech Laboratories, CA, USA) by subcloning the full-length cDNA, which was obtained by PCR amplification using differentiated adipocytes as template and the following primers: PPARγ2 forward:5′-CGA ATT CGG TCG ACC ATG GGT GAA ACT CTG GGA GAT TCT-3′ and reverse:5′-GCG GTA CCT CGA GCT AAT ACA AGT CCT TGT AGA TCT C-3′; RXRα forward:5′-CGA ATT CGG TCG ACC ATG GCT GTC CCC TCG CTG CAC CCC TCC TTG GGT-3′ and reverse:5′-GCA TGC GGC CGC GGT CTA GGT GGC TTG ATG TGG TGC CTC CAG CAT-3′. pcDNA3.1-lacZ (Thermo Fisher Scientific, USA) was used to evaluate transfection efficiency.

COS7 cells were seeded in 12-well plates and cultured in DMEM containing 1.0 g/L D-glucose, 10% FBS, 100 U/mL penicillin, 0.1 mg/mL streptomycin at 37 °C in a 5% CO_2_ incubator. The next day, the cells were exposed to a transfection mixture containing 350 ng of reporter plasmid, 100 ng of pcDNA3.1-lacZ, 50 ng of pCMV-PPARγ2, 50 ng of pCMV-RXRα, 1 μL of Lipofectamine 3000 (Thermo Fisher Scientific), and 2 μL of P3000 reagent (Thermo Fisher Scientific) in 100 μL of serum-free OptiMEM (Thermo Fisher Scientific, USA) in each well. Twenty-four hours after transfection, cells were treated with mifepristone or a PPARγ agonist, pioglitazone, with or without a PPARγ antagonist, T00709007, and incubated for an additional 24 h. Cells were then lysed and subjected to a luciferase activity assay using the luciferase assay system (E4550, Promega, WI, USA). β-Galactosidase activity was determined using a chromogenic substrate, o-nitrophenyl-beta-D-galactopyranoside (ONPG) (Thermo Fisher Scientific). The luciferase activity was normalized to the corresponding β-galactosidase activity.

### DNA microarray analysis

Total RNA was isolated from 3T3-L1 cells as previously reported [14]. The synthesis of the target cRNA probes and hybridization were performed by Macrogen (Macrogen Inc., Seoul, Korea) using the Clariom™ S Assay, Mouse (Thermo Fisher Scientific Affymetrix, Santa Clara, California, USA). Transcriptome Analysis Console (TAC) 4.0, from ThermoFisher Scientific was used to analyze Expression Array feature intensity (CEL) files. The analysis was carried out using the Clariom_S_Mouse NetAffx Library. The microarray gene expression profiles of normal mouse adipose tissues (GSE109371, GSE145750 and GSE150162) were obtained from the NCBI Gene Expression Omnibus (GEO) database (http://www.ncbi.nlm.nih.gov/geo). A statistical analysis to detect differentially expressed genes (DEGs) was carried out with the default settings of TAC, except that the false discovery rate (FDR) p-values were set from false to true. Genes that were significantly upregulated or downregulated by more than twofold (according to a *t*-test) in comparison to mifepristone and exhibited a p-value of < 0.05 and FDR < 0.01 were selected as DEGs, which were subjected to a gene ontology (GO) analysis using Metascape (http://metascape.org). The microarray expression data files were deposited in the Sequence Read Archive (DRA) of DNA Data Bank of Japan (DDBJ). The expression dataset of genes obtained was deposited in the DDBJ Genomic Expression Archive (GEA) with the Experiment Accession number E-GEAD-632.

### Animal experiments

All studies were approved by the Kagawa University Animal Care and Use Committee (IACUC). Seven-week-old C57BL/6NCr Slc male mice were purchased from SLC (Shizuoka, Japan). The animals were handled in compliance with the guidelines of the Animal Experiment at Kagawa University. Mice were housed, at one mouse per cage, at 25 °C and in a 12-h light/12-h dark cycle. The mice were acclimated for one week with ad libitum access to a normal regular diet (RD) (Oriental MF diet, Oriental Yeast. Co., Tokyo, Japan) and water. They were then divided into four groups: the control group (vehicle) and groups treated with mifepristone at 0.1, 1 and 30 mg/kg/day. The mice were maintained for 12 weeks. RD containing an appropriate amount of mifepristone was administered, which was calculated based on the daily consumption of RD (approximately 2 g/day). The dose of mifepristone was chosen according to a previous report [17].

### Statistical analysis

The results are expressed as the mean ± S.E.M. of the indicated numbers of independent experiments. The data were first subjected to the evaluation of the normality using the Shapiro–Wilk test provided in SPSS ver. 28 software (IBM, Tokyo, Japan). For the data that showed the normality, statistical significance was evaluated among multiple groups using a one-way ANOVA followed by Tukey’s multiple comparisons test on Prism ver.6 (GraphPad, San Diego, CA, USA). The data that failed to show the normality were subjected to a non-parametric Kruskal–Wallis test followed by a Dunn’s multiple comparison test on Prism ver.6. In some experiments, an unpaired Student’s t-test was performed to analyze the statistical significance of the difference between the two groups. *P* values of < 0.05 were considered to indicate statistical significance.

## Results

### Enhancement of adipocyte differentiation by mifepristone in 3T3-L1 cells

After reaching confluence, 3T3-L1 cells were differentiated into mature adipocytes according to the conventional protocol, which consisted of 2-day treatment with a mixture of insulin, dexamethasone, and IBMX, followed by 3-day treatment with insulin [15] (Fig. 1A). 3T3-L1 cells were reported to undergo spontaneous adipocyte differentiation when maintained at confluence [18]. However, in present study, continuous culture of 3T3-L1 cells after reaching confluence without inducing adipocyte differentiation caused a significant loss of viability, and the cells eventually detached from the culture dish. The addition of 1 µM mifepristone during a 5-day incubation with the differentiation medium significantly enhanced lipid accumulation (Fig. 1B). Mifepristone had no significant effect on the increase in cell numbers at least until 6 days after the induction of adipogenesis (Fig. 1C). Cell counting was halted thereafter due to technical interference with lipid accumulation; however, the cell density observed with mifepristone appeared comparable to that observed without mifepristone on day 12 when lipid accumulation was evaluated (Fig. 1B). The enhancement of Bodipy staining by mifepristone does not seem to be attributable to an increase in cell number.

Adiponectin was undetectable in 3T3-L1 cells before the induction of adipogenesis, whereas its expression progressively and significantly increased on days 5 and 8 (Fig. 1D). This increase in the adiponectin expression was significantly augmented by treatment with 1 µM mifepristone (Fig. 1D). Mifepristone, in a concentration-dependent manner, enhanced the mRNA expression of adiponectin, Fabp4, and PPARγ2 (adipocyte-specific isoform of PPARγ) but not PPARγ on day 5, all of which are markers of adipocyte differentiation [19, 20] (Fig. 1E). This augmenting effect was observed with 1 µM mifepristone; however, 10 µM mifepristone reduced or had no significant effect (Fig. 1E). The PPARγ agonist pioglitazone has been reported to induce adipogenesis and upregulate the adiponectin expression in 3T3-L1 cells [21–23]. Indeed, both pioglitazone and mifepristone enhanced the expression of adiponectin protein on day 5 after the induction of adipogenesis using the conventional protocol (Fig. 1F). The augmenting effect of 1 µM pioglitazone (30-fold increase) was much greater than that of 1 µM mifepristone (sixfold increase).

### Critical period of mifepristone treatment for enhancement of adipocyte differentiation in 3T3-L1 cells

The critical period for mifepristone treatment to augment the expression of adiponectin and Fabp4 mRNA (Fig. 2B) and protein (Fig. 2C) in 3T3-L1 cells was determined according to the protocols shown in Fig. 2A. When mifepristone treatment was started on day 0 (Fig. 2A, protocols 2–5), the levels of adiponectin and Fabp4 mRNA and proteins progressively increased, depending on the length of mifepristone treatment, and the levels of protein, but not mRNA, of adiponectin and Fabp4 reached levels significantly higher than the control levels (protocol 1) according to protocols 4 and 5. When mifepristone treatment was started on day 2 (protocols 6–8), the levels of adiponectin and Fabp4 mRNA and proteins progressively increased depending on the length of mifepristone treatment; however there was no statistically significant increases in either mRNA or protein levels of adiponectin or Fabp4, even in protocol 8 (Fig. 2B, C). Mifepristone treatment for the last five days (protocol 9), but not the last two days (protocol 10), significantly increased the levels of mRNA, but not protein, of adiponectin and Fabp4 (Fig. 2B, C). The mRNA expression of PPARγ2 showed similar changes to those seen with adiponectin and Fabp4; however, there were no statistically significant changes in PPARγ2 (Fig. 2B).

### Paracrine/autocrine effect of adiponectin in the mifepristone-induced enhancement of adipocyte differentiation in 3T3-L1 cells

Adiponectin has been reported to enhance the adipogenesis of 3T3-L1 cells in a paracrine and autocrine manner [24, 25]. To investigate the involvement of this mechanism in the enhancement of adipogenesis by mifepristone, the effect of mifepristone on adiponectin secretion were examined (Fig. 3). Fetal bovine serum (FBS) contains bovine adiponectin, which interferes with the determination of adiponectin secretion from mouse 3T3-L1 cells. Therefore, specimens were obtained in serum-free medium according to the protocol shown in Fig. 3A. In the vehicle control group, no significant secretion of adiponectin was observed on days 6, 7, or 8 (Fig. 3A). Treatment with mifepristone significantly enhanced adiponectin secretion in a time-dependent manner (Fig. 3A). Moreover, treatment with mifepristone during the first two days after the induction of adipogenesis significantly enhanced the subsequent secretion of adiponectin on days 4 and 5 (Fig. 3B).

The role of secreted adiponectin in adipogenesis was investigated by examining the effects of the adiponectin-neutralizing antibody, ANOC 9104 [26, 27], on mifepristone-induced enhancement of adipogenesis. The neutralizing antibody (12.5 µg/mL) was applied from day 0 to day 12, according to the protocol shown in Fig. 1A. The enhancement of lipid accumulation by mifepristone, as evaluated by Bodipy 493/503 staining, was significantly attenuated by treatment with the neutralizing antibody (Fig. 3C). The neutralizing antibody itself had little effect on the lipid accumulation observed without mifepristone treatment. The increase in the expression of adiponectin and Fabp4 mRNA and protein observed with mifepristone treatment was significantly, but partly, inhibited by treatment with the neutralizing antibody (Fig. 3D and E).

PPARγ is a well-known master regulator of adipogenesis [28, 29]. Mifepristone has been reported to exert a partial agonistic effect on PPARγ [13, 14]. Co-treatment with 10 μM T0070907, a PPARγ antagonist, abolished mifepristone-induced upregulation of Fabp4 and PPARγ mRNA on day 5 after the induction of adipogenesis (Fig. 3F). T0070907 also significantly inhibited the increase in the adiponectin protein expression that was induced by mifepristone (Additional file 1: Fig. S1B). Culture in the presence of serum has been reported to induce adipocyte differentiation in 3T3-L1 [30–32]. It is possible that mifepristone induced adipocyte differentiation in cooperation with serum. Therefore, the adipocyte-differentiating effect of mifepristone was investigated under serum-free conditions. After reaching confluence, the cells were deprived of serum and treated with mifepristone. A large part of the cells became detached from the substratum five days after incubation under serum-free conditions, both with and without mifepristone treatment. In the remaining attached cells, the mRNA expression of Fabp4 and PPARγ2, but not adiponectin or PPARγ, significantly increased (Additional file 1: Fig. S2). However, the formation of lipid droplets was not observed 10 days after the addition of mifepristone.

### Induction of adipocyte differentiation by mifepristone alone in 3T3-L1 cells

The observation of mifepristone-induced enhancement of adipocyte differentiation by the conventional protocol prompted the investigation to determine whether mifepristone alone is capable of inducing adipocyte differentiation in 3T3-L1 cells. Lipid accumulation, as evaluated by Bodipy 493/503 staining, was increased in a concentration-dependent manner by mifepristone alone on day 12, while a significant increase was observed with 5 and 10 μM (Fig. 4A). The expression of adiponectin protein was also significantly increased by 5 and 10 μM mifepristone on day 6 (Fig. 4B). Lipid accumulation and the upregulation of adiponectin protein induced by mifepristone were inhibited by co-treatment with the PPARγ antagonist T0070907 and the adiponectin-neutralizing antibody, while those induced by pioglitazone, a PPARγ agonist, were also inhibited by T0070907 (Additional file 1: Fig. S1A and S1B). Moreover, antagonists of other steroid hormone receptors, including levonorgestrel for progesterone receptor, eplerenone for aldosterone receptor, and spironolactone for mineralocorticoid receptor, had no effect on the adiponectin expression (Additional file 1: Fig. S1B). Similarly, progesterone or estradiol, steroid hormone receptor agonists, also had no effect on adiponectin expression (Additional file 1: Fig. S1B).

### Role of PPARγ in the mifepristone-induced adipocyte differentiation in 3T3-L1 cells

Transfection of siRNA targeting mouse PPARγ substantially suppressed the expression of PPARγ mRNA compared to that seen with control siRNA, both 2 days and 7 days after transfection (Fig. 5A, left panel). The significant suppression of the protein expression of PPARγ, but not Rac1, was also observed 7 days after transfection (Fig. 5A, right panel). The increase in the expression of adiponectin and Fabp4 induced by mifepristone alone was abolished when the expression of PPARγ was knocked down (Fig. 5B).

### Activation of the promoter activity of the PPAR response element by mifepristone

The effect of mifepristone on the activity of the promoter containing the PPAR response element was examined in COS7 cells using a luciferase reporter assay (Fig. 6). When COS7 cells were transfected only with reporter plasmids for firefly luciferase and β-galactosidase, mifepristone (10 μM) had no significant effect on the promoter activity (Fig. 6A, conditions 1 and 3). Pioglitazone (1 μM), used as a positive control, significantly increased the promoter activity under the same conditions (Fig. 6A, condition 2). When COS7 cells were co-transfected with a PPARγ2 expression plasmid (Fig. 6A, conditions 4–7), 1 μM—but not 10 μM—mifepristone significantly increased promoter activity. The effect of pioglitazone on the promoter activity was also enhanced in the presence of the PPARγ2 expression plasmid. The co-expression of PPARγ2 and RXRα further augmented the promoter activity obtained with both 1 and 10 μM mifepristone (Fig. 6A, conditions 8–11). However, no further increase in promoter activity was observed with pioglitazone treatment. Therefore, the concentration-dependent effect of mifepristone on promoter activity was examined under these conditions, that is, the co-expression of PPARγ2 and RXRα (Fig. 6B). The maximal effects of mifepristone were observed at 3 μM (Fig. 6B). The promoter-activating effects of mifepristone and pioglitazone were completely abolished by treatment with PPARγ antagonist T0070907 (Fig. 6A, conditions 12–15).

### Characterization of mifepristone-differentiated adipocytes by a DNA microarray analysis

**Fig. 7:**
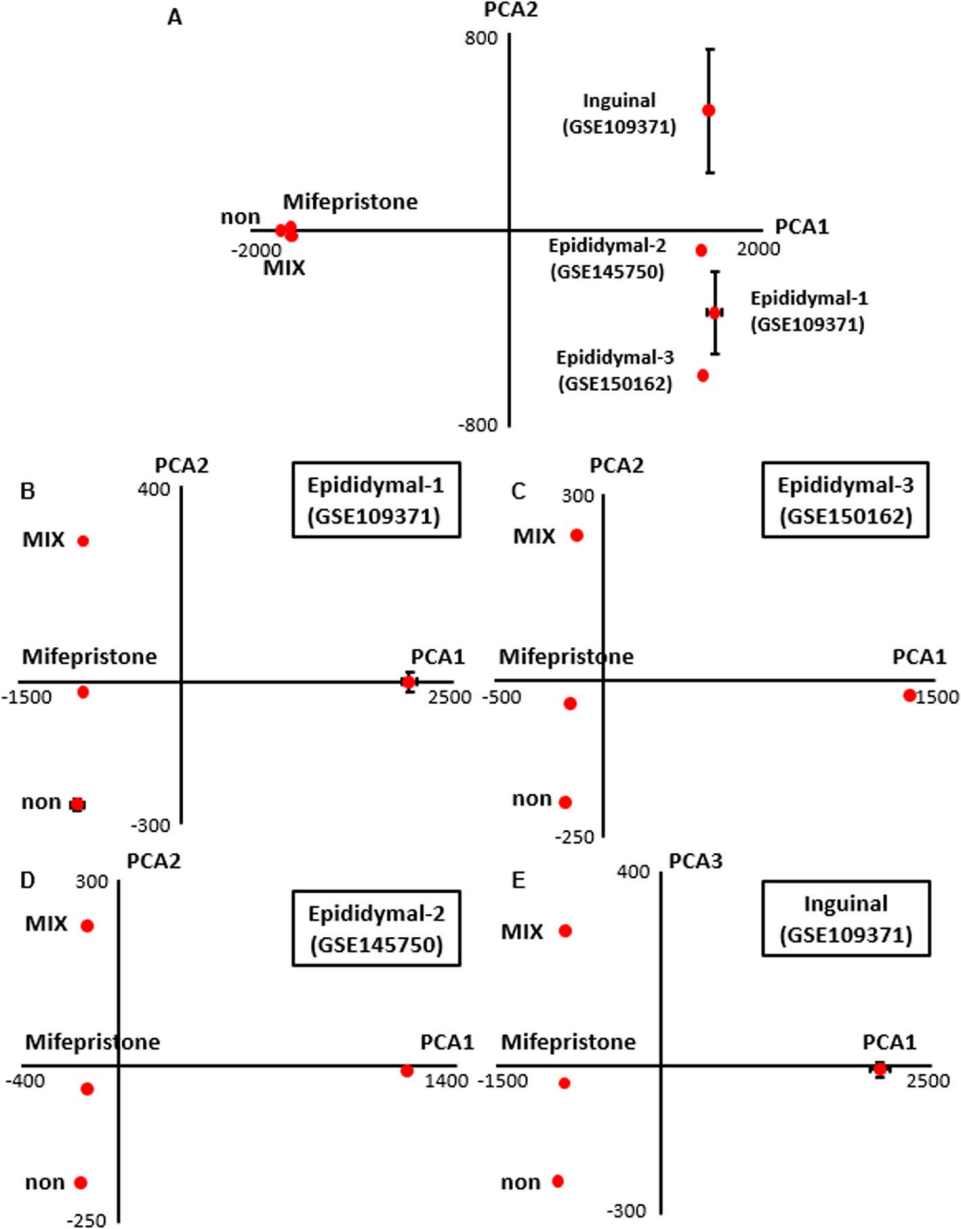
The principal component analysis of three datasets of 3T3-L1 cell and four registered datasets of the normal mouse adipose tissues. The principal component analysis of the datasets of 3T3-L1 cells under three conditions (non, mifepristone, and MIX), as described in the Materials and Methods section, with all registered datasets of epidydimal or inguinal adipose tissues (**A**), or any one of the four registered datasets as indicated (**B**–**E**)

A principal component analysis (PCA) with all datasets (i.e., the datasets obtained in the present study from 3T3–L1 cells under three different conditions (non-adipogenic control condition [non] and the adipogenic conditions with the conventional protocol [MIX] and the single treatment with mifepristone), and those obtained from the NCBI database for inguinal (GSE109371) and epididymal (GSE109371, GSE145750, GSE150162) adipose tissues of normal mice, revealed clear differences between 3T3–L1 cells and adipose tissues and proximity among the three datasets of 3T3-L1 cells (Fig. 7A, Additional file 2: Table S1). The datasets of mouse adipose tissues were similar to each other in terms of PCA1 but differed in terms of PCA2 (Fig. 7A). When the three datasets of 3T3-L1 cells and one of the four datasets of mouse adipose tissues were analyzed, the dataset of the mifepristone-differentiated adipocytes showed proximity to all adipose tissues in terms of either PCA2 or PCA3, while the datasets of 3T3-L1 cells showed proximity to each other and distance from the dataset of the adipose tissues in terms of PCA1 (Fig. 7B–D).

**Fig. 8:**
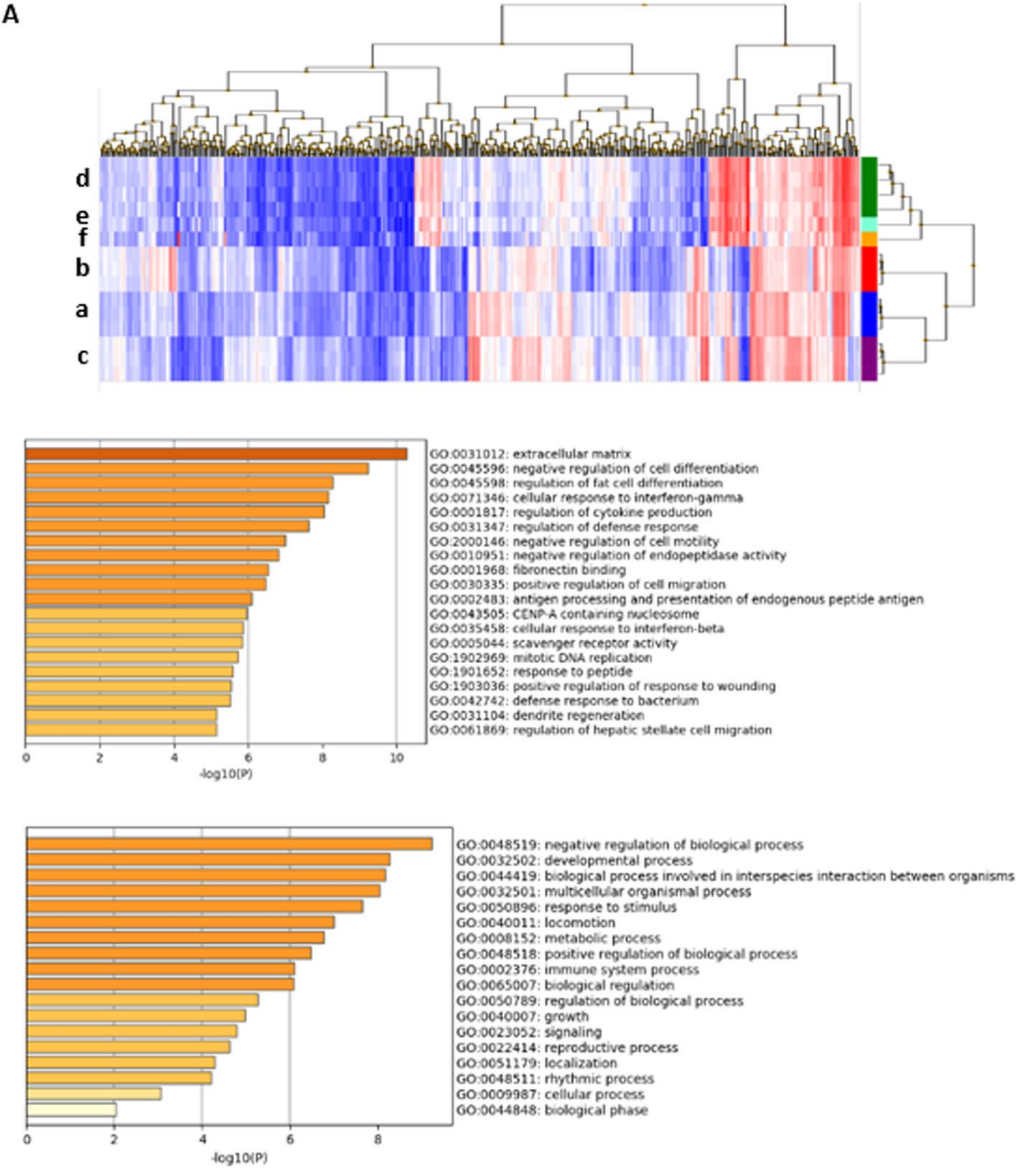

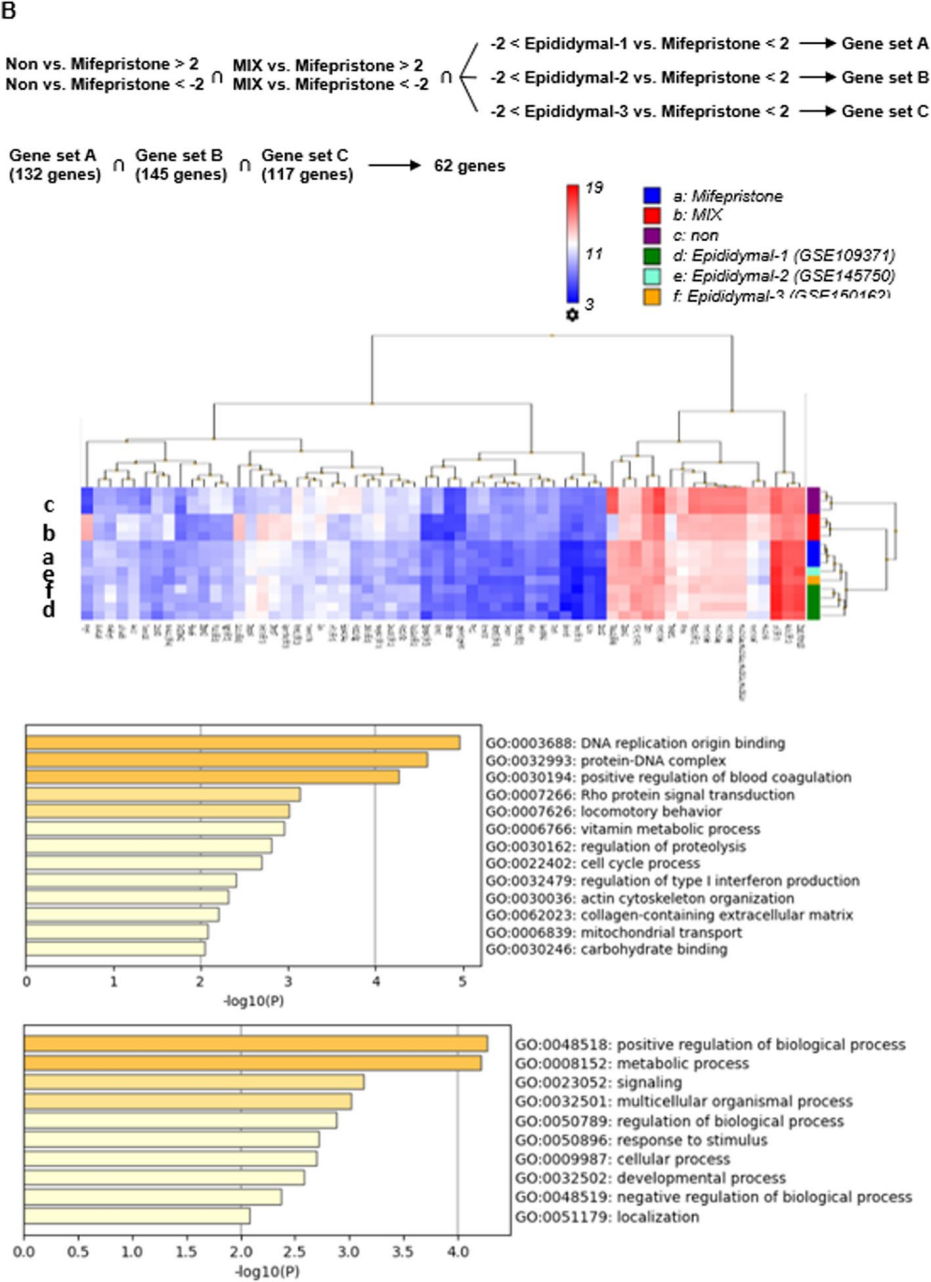
Hierarchical clustering and the gene ontology (GO) enrichment analysis of the differentially expressed genes (DEGs). Hierarchical clustering display of the datasets (a–f as indicated in a key) and the GO enrichment analysis of 403 transcripts that showed a significant twofold difference in expression level between mifepristone-differentiated adipocytes (a, mifepristone) and adipocytes differentiated by the conventional differentiation protocol (b, MIX) or non-differentiated adipocytes (c, non) (**A**) and 62 genes that were selected by the algorithm shown on the top (**B**). Namely, 62 genes were differentially expressed (> twofold) between mifepristone (a) vs. MIX (b) and non (b), but commonly expressed (< twofold) in mifepristone (a) and either one of epididymal adipose tissues (d, e, f). The top GO terms with a value of -log_10_P > 2 in all three subcategories (top bar graph) and a subcategory of biological processes (lower bar graph) are shown below the hierarchical clustering display

The extraction of DEGs common to those between the mifepristone-differentiated adipocytes and the adipocytes differentiated by the conventional protocol and those between the mifepristone-differentiated adipocytes and the non-differentiated control 3T3-L1 cells (non) yielded 403 genes at a significance threshold of *P* < 0.01 (Fig. 8A, left panel; Additional file 2: Table S2). A GO enrichment analysis of 403 DEGs with Metascape revealed enrichment in the regulation of the extracellular matrix (GO: 0031012), negative regulation of cell differentiation (GO:0045596), regulation of fat cell differentiation (GO:0045598), cellular response to interferon-gamma (GO:0071346), and regulation of cytokine production (GO:0001817) (Fig. 8A, right upper panel). In terms of the biological process (BP) subcategories, enrichment in negative regulation of biological processes (GO: 0048519), developmental processes (GO:0032502), biological processes involved in interspecies interactions between organisms (GO:0044419), and multicellular organismal processes (GO:0032501) were obtained.

Among the 403 genes, 62 were found to be common between the mifepristone-differentiated adipocytes and any of the three epididymal adipose tissues (Fig. 8B, left panel; Additional file 2: Table S3). These genes are considered to be related to the characteristics of mifepristone-differentiated adipocytes, which are similar to those in in situ adipose tissues. GO terms were enriched for the regulation of DNA replication origin binding (GO: 0003688), protein-DNA complex (GO: 0032993), positive regulation of blood coagulation (GO: 0030194), and Rho protein signal transduction (GO:0007266). In terms of the BP subcategories, enrichment in the positive regulation of biological processes (GO: 0048518), metabolic processes (GO: 0008152), signaling (GO: 0023052), and multicellular organismal processes (GO: 0032501) were obtained.

### In vivo effects of mifepristone on the development of adipose tissues in mice

**Fig. 9:**
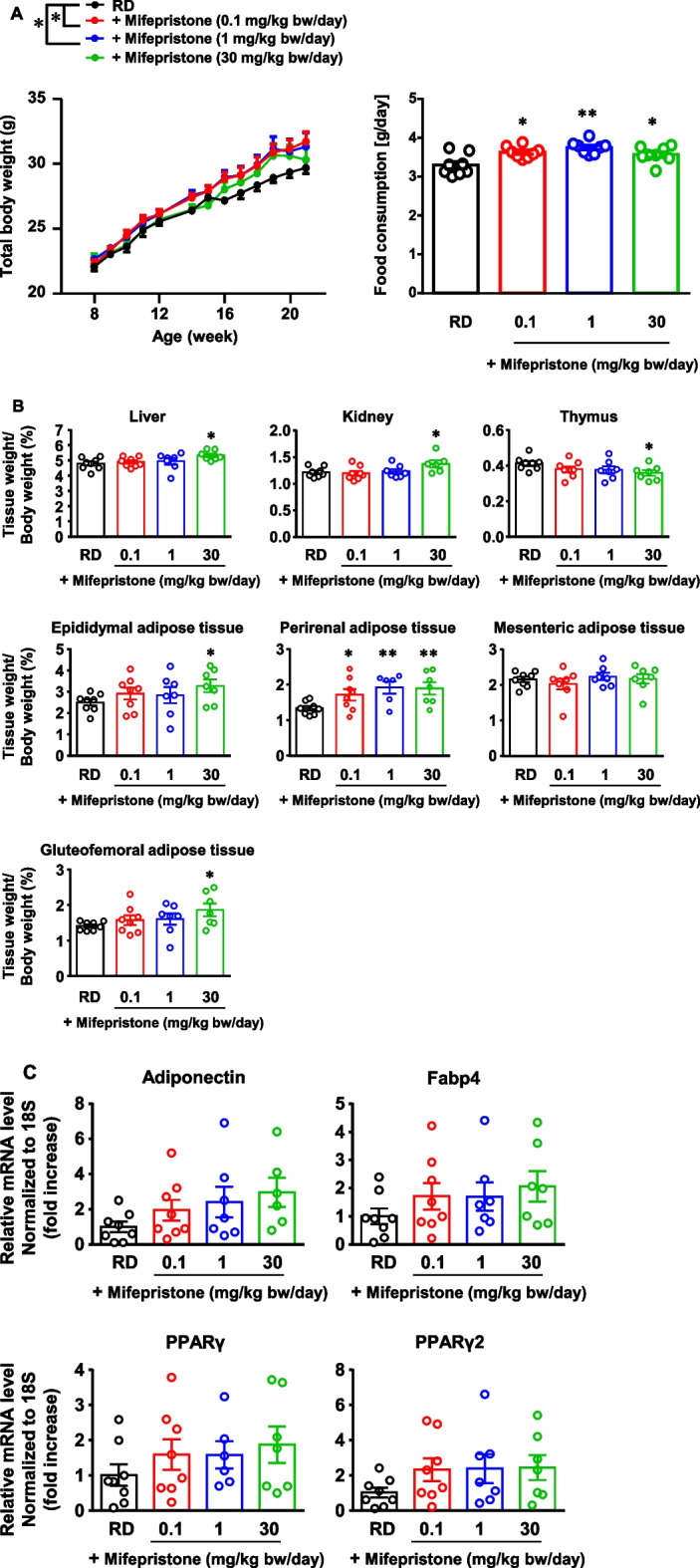
In vivo adipogenic effect of mifepristone in mice. **A** Summary (*n =* 8) of the dose-dependent effects of mifepristone (0.1, 1, and 30 mg/kg bw/day) on the time course of changes in the body weight after 12-week oral treatment with mifepristone. The summary of the calculated average daily food consumption during the experimental protocol (8–20 weeks) in the indicated experimental groups. The mice were fed a regular diet (RD) with or without mifepristone for 12 weeks. **B** Summaries of the body weight-normalized weights of the indicated organs and tissues. **C** Summaries of the mRNA expression of adiponectin, Fabp4, PPARγ and PPARγ2 in epididymal adipose tissues obtained from the mice of the indicated experimental groups. The mRNA levels were normalized by that of 18S rRNA and expressed as a fold increase from those of the mice of RD. The data represent the mean ± S.E.M. *P < 0*.*05; **P < 0*.*01 vs. RD

The in vivo adipogenic effects of mifepristone were investigated by treating 8-week-old mice with mifepristone for 12 weeks. Mifepristone treatment augmented the increase in body weight, whereas treatment with 0.1 and 1 mg/kg body weight/day resulted in a significantly greater body weight in comparison to the control group (Fig. 9A). Mifepristone treatment modestly but significantly increased the food consumption (Fig. 9A). Part of the increase in body weight gain by mifepristone may be due to the increase in food consumption. Mifepristone treatment significantly increased the body weight-normalized weight of the liver, kidneys, and epididymal, perirenal, and gluteofemoral adipose tissues but not the mesenteric adipose tissues (Fig. 9B). The weight of the thymus decreased after mifepristone treatment (Fig. 9B). Mifepristone treatments appeared to upregulate the mRNA expression of adiponectin, Fabp4, PPARγ and PPARγ2 in epididymal adipose tissues; however, there were no statistically significant differences (Fig. 9C).

## Discussion

The most noteworthy finding of the present study was that mifepristone alone was capable of inducing adipocyte differentiation in 3T3-L1 cells. However, full adipocyte differentiation induction by mifepristone requires the presence of certain factors in the serum used in the cell culture. Mifepristone also enhanced the adipocyte differentiation induced by a conventional protocol. Mechanistically, PPARγ plays a critical role in mifepristone-induced adipocyte differentiation because the pharmacological and genetic inhibition of PPARγ inhibits the induction of adipocyte differentiation markers, such as adiponectin and Fabp4. Mifepristone has been reported to function as a partial PPARγ agonist [13, 14]. Indeed, mifepristone activated the promoter activity of PPRE in a PPARγ expression-dependent manner and was sensitive to the PPARγ inhibitor. Thus, it is hypothesized that mifepristone activates PPARγ, thereby inducing adipocyte differentiation in 3T3-L1 cells. Furthermore, the primary component analysis of the DNA microarray data suggested that the mifepristone-induced adipocytes may represent some characteristics of the in situ adipocytes to a greater extent than those induced by the conventional protocol. Therefore, we propose a single treatment with mifepristone in the presence of serum as a novel protocol to induce more physiologically relevant adipocytes in 3T3-L1 cells than the conventional protocol.

It is somewhat counterintuitive that mifepristone induced adipocyte differentiation because mifepristone exerts an antagonistic effect on the glucocorticoid receptor, which contributes—in part—to adipocyte differentiation by the conventional protocol [6, 8]. The Kd value of mifepristone for the glucocorticoid receptor was reported to be 0.4–0.5 nM [9–12], while the EC50 values for agonistic effect of mifepristone on PPARγ is estimated to be 0.1 μM according to the promoter assay using PPRE [13]. Furthermore, in silico molecular modeling estimated the docking affinity of mifepristone for PPARγ and glucocorticoid receptor to be − 9.3 kcal/moL [33] and − 15.75 kcal/moL (http://repositorio.colciencias.gov.co/handle/ 11,146/34221), respectively. Mifepristone is thus considered to exert an antagonistic effect on glucocorticoid receptors at nanomolar concentrations and an agonistic effect on PPARγ at micromolar concentrations. The present study showed that the optimum concentration of mifepristone to induce adipocyte differentiation was 3 μM. It is hypothesized that the PPARγ-mediated adipocyte differentiation effect of mifepristone dominates its antagonistic effect on glucocorticoid receptors at micromolar concentrations. However, at concentrations higher than 3 μM, mifepristone exhibited a lower effect on adipocyte differentiation (Figs. 1E and 4B), which was consistent with a lower effect on the activation of PPARγ-dependent promoter activity (Fig. 6A). These observations provide additional support for the dependence of mifepristone-induced adipocyte differentiation on PPARγ agonistic effects. PPAR agonists such as pioglitazone, which was used as a positive control for mifepristone, have been reported to induce adipocyte differentiation [19, 34]. The present study also demonstrated that pioglitazone induced the expression of adiponectin and lipid deposition. The usefulness of PPARγ in inducing adipocyte differentiation is consistent with the notion that PPARγ contributes to the late phase of the terminal differentiation of adipocytes and maintenance of the mature adipocyte phenotype.

The balance between the antagonistic effect on glucocorticoid receptor and the agonistic effect on PPARγ activity is suggested to determine the overall effect of mifepristone, especially when added to the conventional protocol. In cases where the induction of adiponectin by mifepristone was dependent on the agonistic effect on PPARγ, there was an apparent inconsistency between the level of adiponectin and PPARγ (Fig. 1E). The level of PPARγ obtained with 10 μM mifepristone was twofold that of the control level, although the increase was insignificant, while the level of adiponectin obtained with 10 μM mifepristone was similar to the control level. The agonistic effect of mifepristone on PPARγ at 10 μM was lower than that seen with 1 μM (Fig. 6B). In line with this, the induction of adiponectin by mifepristone was lower at 10 μM than at 3 μM (Fig. 4B). The decrease in the adiponectin level seen with 10 μM mifepristone during the conventional protocol is speculated to be due to the antagonistic effect of mifepristone on the glucocorticoid receptor-mediated induction of adiponectin and the decrease in the agonistic effect on PPARγ.

In addition to the optimal concentrations of mifepristone, there seems to be a critical period of mifepristone treatment to enhance adipocyte differentiation using the conventional protocol, which depends on both the specific timing and length of treatment. Three days of treatment was insufficient; a ≥ 5-day treatment period was needed. The 5-day treatment was less effective when the mifepristone treatment overlapped with a part or a whole of the 5-day conventional protocol treatment. This reduced effect on adipocyte differentiation might be due to the antagonistic effect of mifepristone on glucocorticoid receptors. Furthermore, 5-day treatment was insufficient to induce adiponectin protein expression. Therefore, ≥ 8-day treatment with mifepristone is suggested to be optimal for inducing adipocyte differentiation.

Adiponectin not only serves as an adipocyte differentiation marker but also plays a more functional role during adipocyte differentiation. As the addition of the adiponectin-neutralizing antibody prevented lipid accumulation and the expression of adipocyte markers, adiponectin contributed to adipocyte differentiation via an autocrine or paracrine mechanism. This functional role of adiponectin is consistent with that reported in previous studies [35, 36]. The expression of adiponectin was induced after 2 days of the conventional protocol, and the addition of adiponectin enhanced adipocyte differentiation. Adiponectin is an adipokine secreted from adipocytes that exerts more physiological but not pathological functions [37–39]. Adiponectin has been reported to induce adipocyte differentiation via PPARγ [35, 36]. PPARγ is a key regulator of physiological adipogenesis [5, 40, 41], whereas the adiponectin gene is one of the genes regulated by PPARγ [7]. Therefore, the greater contribution of adiponectin and PPARγ to mifepristone-induced adipocyte differentiation may be related to the fact that the mifepristone-induced adipocytes were closer to in situ adipocytes than those induced by the conventional protocol.

The findings of the present study also suggest a role of RXRα in the adipocyte differentiation induced by mifepristone. The involvement of RXRα is consistent with previous reports showing that the expression of both RXRα and PPARγ was upregulated during adipocyte differentiation during the conventional protocol in 3T3-L1 cells and that the RXRα agonist alone induced adipocyte differentiation in 3T3-L1 cells [42]. However, there is no evidence to support that mifepristone acts directly on RXRα. Furthermore, the adipocyte differentiating effect of mifepristone is independent of the receptors of estrogen, progesterone, or mineral corticoids. Mifepristone-induced promoter activity was completely inhibited by the PPARγ inhibitor, even in the presence of RXRα. PPARγ appears to be the primary target of mifepristone.

The 3T3-L1-derived adipocytes induced by either mifepristone or the conventional protocol were still closer to each other and were quite different from the in situ adipocytes with respect to the PCA1 gene profile. However, the increase in the weight of epididymal, perirenal and gluteofemoral adipose tissues may support the in vivo adipogenic effect of mifepristone. This observation is inconsistent with our previous report that mifepristone prevents high-fat diet-induced adipocyte hypertrophy and reduces the expression and secretion of adipocytes [14]. The mechanism of pathologic adipogenesis observed in obesity is different from that of the physiological development of adipose tissues [43]. Glucocorticoids and proinflammatory cytokines contribute to pathological adipogenesis [44–46]. Mifepristone might have inhibited adipocyte hypertrophy by antagonizing the effects of glucocorticoids as a glucocorticoid receptor antagonist or by inducing adiponectin as a PPARγ agonist in the high-fat diet-fed mice. Overall, mifepristone may contribute to the development of physiological adipocytes.

Regarding our proposal that mifepristone induces physiologically relevant adipocyte differentiation in 3T3-L1 cells, it should be noted that 3T3-L1 cells are not capable of forming adipose tissues in vivo when implanted subcutaneously in thymectomized mice [47]. In contrast, 3T3-F442A cells, sister of 3T3-L1 cells, do form adipose tissues in vivo under such conditions. This fact might limit the translation of our findings obtained with 3T3-L1 cells to adipogenesis in vivo. The development of mature adipose tissues appears to require some factors that might be missing in 3T3-L1 cells in addition to the terminal differentiation of preadipocytes to adipocytes at the cellular level. Our findings concerning the characteristics of the gene expression pattern of the mifepristone-induced adipocytes that were closer to those seen with the in situ adipose tissues are considered to represent the capability of mifepristone for inducing terminal differentiation.

In conclusion, the findings of the present study propose a novel method to induce adipocyte differentiation in 3T3-L1 cells by a single treatment with mifepristone at micromolar concentrations for > 5 days. Notably, mifepristone-induced adipocytes are closer to the in situ adipocytes of normal adipose tissues than those induced by the conventional protocol. Thus, the mifepristone treatment protocol is useful for investigating adipocytes under conditions closer to the physiological in situ situation.

### Supplementary Information

**Additional file 1: Figure S1.** Effects of mifepristone, pioglitazone and other steroid receptor agonists or antagonist on adipocyte differentiation in 3T3 L1 cells. **A** Representative fluorescent microscopic images of Bodipy 493/503 (green) fluorescence on day 10 after treatment with 1 μM mifepristone or 1 μM pioglitazone either in the presence or absence of PPARγ antagonist (1 μM T0070907) and adiponectin-neutralizing antibody (12.5 ng/mL ANOC9140), as indicated. **B** The effect of mifepristone, pioglitazone and other steroid receptor agonists or antagonist on the expression of adiponectin after 6-day treatment. **Figure S2.** Effects of mifepristone under serum-free conditions on adipocyte differentiation in 3T3 L1 cells. (Upper) Summaries (*n =* 6) showing the effect of 1 μM mifepristone on the mRNA expression of Fabp4, adiponectin, PPARγ, and PPARγ2 after 5-day treatment. (Lower) Representative fluorescent microscopic images and summary (*n =* 3) of quantitative evaluation of Bodipy 493/503 (green) fluorescence after 10-day treatment with 1 μM mifepristone under serum-free conditions. The data represent the mean ± S.E.M. *P < 0.05; ns, not significantly different.

**Additional file 2: Table S1.** List of series accession numbers of the GEO data sets of in situ mice adipose tissues obtained with Clariom_S_Mouse DNA chip (Platoform: GPL23038) and deposited on NCBI Gene Expression Omnibus (GEO) database (http://www.ncbi.nlm.nih.gov/geo). **Table S2.** Differentially expressed genes (403 DEGs) that are common to those between the mifepristone-differentiated adipocytes and the adipocytes differentiated by the conventional protocol and those between the mifepristone-differentiated adipocytes and the non-differentiated control 3T3-L1 cells. **Table S3.** The genes (62 genes) among 403 DEGs (Table S2) that are common between the mifepristone-differentiated adipocytes and any of the three epididymal adipose tissues.

## Data Availability

All data generated or analyzed during this study are included in this published article and its Additional files. The raw data supporting the conclusions of this manuscript will be made available by the authors without undue reservation to any qualified researcher.

## Abbreviations

PPARγ: Peroxisome Proliferator-Activated Receptor-γ
Fabp4: Adipocyte fatty acid binding protein-4
PPRE: PPAR-response element
PCA: Principal component analysis
IBMX: Isobutyl-Methylxanthine
CREB: Cyclic AMP-Response Element Binding Protein
qRT-PCR: Real-time quantitative RT-PCR
NAb: Neutralizing Antibody for adiponectin
DMEM: Dulbecco’s Modified Eagle’s Medium
INS: Insulin
PBS: Phosphate-Buffered Saline
HRP: Horseradish Peroxidase
siRNA: Small interfering RNA
RXRα: Retinoid X Receptor α
ONPG: O-nitrophenyl-beta-D-galactopyranoside
TAC: Transcriptome Analysis Console
GEO: Gene Expression Omnibus
DEGs: Detect Differentially Expressed Genes
FDR: False Discovery Rate
DDBJ: DNA Data Bank of Japan
DRA: DDBJ Sequence Read Archive
GEA: DDBJ Genomic Expression Archive
GO: Gene Ontology
RD: Regular Diet
FBS: Fetal Bovine Serum
