# Supplementary material for: Effects of mifepristone on adipocyte differentiation in mouse 3T3-L1 cells
Source: Cell Mol Biol Lett. 2024 Mar 29;29:45. doi: 10.1186/s11658-024-00559-9 (PMC10981365; doi:10.1186/s11658-024-00559-9)

**Figure S1.** Effects of mifepristone, pioglitazone and other steroid receptor agonists or antagonist on adipocyte differentiation in 3T3 L1 cells.

(A) Representative fluorescent microscopic images of Bodipy 493/503 (green) fluorescence on day 10 after treatment with 1 μM mifepristone or 1 μM pioglitazone either in the presence or absence of PPARγ antagonist (1 μM T0070907) and adiponectin-neutralizing antibody (12.5 ng/mL ANOC9140), as indicated. (B) The effect of mifepristone, pioglitazone and other steroid receptor agonists or antagonist on the expression of adiponectin after 6-day treatment.


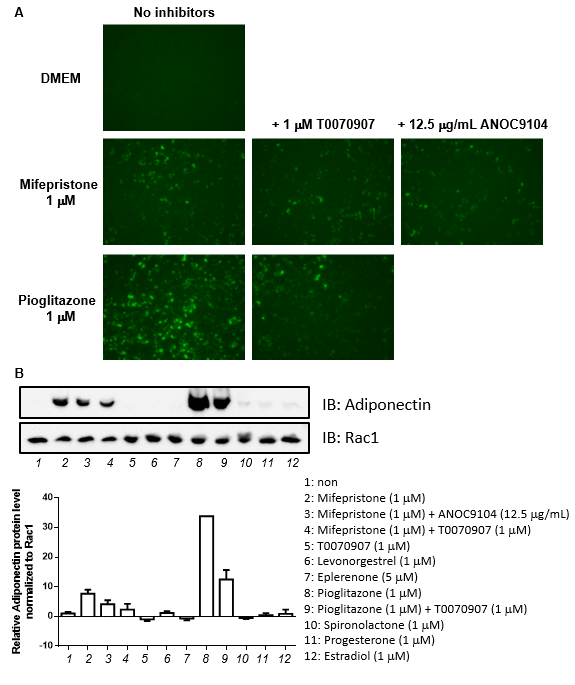


**Figure S2.** Effects of mifepristone under serum-free conditions on adipocyte differentiation in 3T3 L1 cells.

(Upper panel) Summaries (n = 6) showing the effect of 1 μM mifepristone on the mRNA expression of Fabp4, adiponectin, PPARγ, and PPARγ2 after 5-day treatment. (Lower panel) Representative fluorescent microscopic images and summary (n = 3) of quantitative evaluation of Bodipy 493/503 (green) fluorescence after 10-day treatment with 1 μM mifepristone under serum-free conditions. The data represent the mean ± S.E.M. *, P < 0.05; ns, not significantly different.


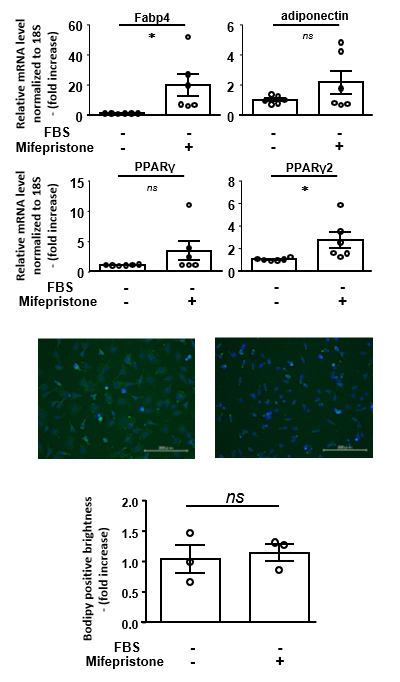

Supplement: Supplementary file 1 — Additional file 1: Figure S1. Effects of mifepristone, pioglitazone and other steroid receptor agonists or antagonist on adipocyte differentiation in 3T3 L1 cells. A Representative fluorescent microscopic images of Bodipy 493/503 (green) fluorescence on day 10 after treatment with 1 μM mifepristone or 1 μM pioglitazone either in the presence or absence of PPARγ antagonist (1 μM T0070907) and adiponectin-neutralizing antibody (12.5 ng/mL ANOC9140), as indicated. B The effect of mifepristone, pioglitazone and other steroid receptor agonists or antagonist on the expression of adiponectin after 6-day treatment. Figure S2. Effects of mifepristone under serum-free conditions on adipocyte differentiation in 3T3 L1 cells. (Upper) Summaries (n = 6) showing the effect of 1 μM mifepristone on the mRNA expression of Fabp4, adiponectin, PPARγ, and PPARγ2 after 5-day treatment. (Lower) Representative fluorescent microscopic images and summary (n = 3) of quantitative evaluation of Bodipy 493/503 (green) fluorescence after 10-day treatment with 1 μM mifepristone under serum-free conditions. The data represent the mean ± S.E.M. *P < 0.05; ns, not significantly different. [file 11658_2024_559_MOESM1_ESM.docx]
