# Supplementary material for: Effects of mifepristone on adipocyte differentiation in mouse 3T3-L1 cells
Source: Cell Mol Biol Lett. 2024 Mar 29;29:45. doi: 10.1186/s11658-024-00559-9 (PMC10981365; doi:10.1186/s11658-024-00559-9)
Supplement: Supplementary file 2 — Additional file 2: Table S1. List of series accession numbers of the GEO data sets of in situ mice adipose tissues obtained with Clariom_S_Mouse DNA chip (Platoform: GPL23038) and deposited on NCBI Gene Expression Omnibus (GEO) database (http://www.ncbi.nlm.nih.gov/geo). Table S2. Differentially expressed genes (403 DEGs) that are common to those between the mifepristone-differentiated adipocytes and the adipocytes differentiated by the conventional protocol and those between the mifepristone-differentiated adipocytes and the non-differentiated control 3T3-L1 cells. Table S3. The genes (62 genes) among 403 DEGs (Table S2) that are common between the mifepristone-differentiated adipocytes and any of the three epididymal adipose tissues. [file 11658_2024_559_MOESM2_ESM.docx]

**Table S1.** List of series accession numbers of the GEO data sets of in situ mice adipose tissues obtained with Clariom_S_Mouse DNA chip (Platoform: GPL23038) and deposited on NCBI Gene Expression Omnibus (GEO) database (http://www.ncbi.nlm.nih.gov/geo).

| Series accession number | Tissues | |
| --- | --- | --- |
| GSE109371 | Epididymal adipose tissue | Inguinal adipose tissue |
| GSE145750 | Epididymal adipose tissue |  |
| GSE150162 | Epididymal adipose tissue |  |

**Table S2.** Differentially expressed genes (403 DEGs) that are common to those between the mifepristone-differentiated adipocytes and the adipocytes differentiated by the conventional protocol and those between the mifepristone-differentiated adipocytes and the non-differentiated control 3T3-L1 cells.

| 1700025G04Rik | Atp6ap1 | Cnn2 | Dusp7 | Fst | Grem1 |
| --- | --- | --- | --- | --- | --- |
| 1810011O10Rik | B3glct | Cntnap1 | E2f8 | Fzd1 | Grik2 |
| 2010016I18Rik | Bcl6 | Col4a1 | Ear12; Ear2; Ear3 | Galnt18 | Grn |
| 2810417H13Rik | Bub1 | Col4a2 | Efhd2 | Gbp4 | Grpr |
| 4930579G24Rik | C1s2 | Col6a3 | Enah | Gbp9 | Gsto2 |
| 9130204L05Rik | C4a | Cryab | Enpp2 | Gbp10; Gbp6 | Gvin1 |
| A4galt | C920025E04Rik | Csprs | Enpp4 | Gbp11 | Gxylt2 |
| Ablim1 | Car6 | Csprs | Enpp5 | Gca | H2afx |
| Acta2 | Car11 | Csprs | Epas1 | Gclc | H2-K1 |
| Acvr2a | Ccbe1 | Csprs | Epb41l4a | Gcnt4 | H2-L; H2-D1 |
| Adamts7 | Ccl2 | Ctla2a | Epha4 | Gins1 | H2-M3 |
| Adipoq | Ccl7 | Ctla2b | Ephx2 | Glis3 | H2-Q2 |
| Adipor2 | Ccl8 | Ctsd; Ifitm10 | Erlin1 | Glul; Mir8114 | H2-Q4 |
| Agrn | Ccl9 | Ctsf | Errfi1 | Gm2016 | Hacd4 |
| Akap12 | Ccl11 | Ctsk | Esco2 | Gm2022 | Hist1h2ae |
| Akr1b7 | Cd36 | Cxcl5 | F830016B08Rik | Gm2046 | Hist1h2af |
| Aldh1a7 | Cdc6 | Cyb561a3 | Fam13a | Gm4951 | Hist1h2ag |
| Aldh1l2 | Cdc45 | Cyth3 | Fam114a1 | Gm5662 | Hist1h2ah |
| Amot | Cdkn2d | D930015E06Rik | Fam198b | Gm7609 | Hist1h2ak |
| Ampd3 | Cebpb | Dap | Fas | Gm8909 | Hist1h2al |
| Angptl4 | Celf2 | Ddias | Fat4 | Gm10715 | Hist1h2ao; Hist1h2ap; Hist1h2ai; Hist1h2ah |
| Ankrd28 | Cenpm | Dennd2d | Fblim1 | Gm18853 | Hist1h2ap |
| Aplp2 | Cenpn | Dhcr7 | Fbln1 | Gm38425; Gab3 | Hist1h3c |
| Apod | Chst15 | Dhfr | Fbxo6 | Gpc6 | Hist1h3e |
| Aqp1 | Ciart | Dhrs1 | Fgf23 | Gpr149 | Hist1h3f |
| Aqp7 | Ckap2 | Dhrs7 | Fgfrl1 | Gpr176 | Hist1h3g |
| Arhgap20 | Clca3a1; Clca1 | Dlx2 | Fhl2 | Gprc5b | Hist1h3h |
| Aspa | Clca3a2; Clca2 | Dqx1 | Fkbp5 | Gprin3 | Hist1h3i |
| Atp2a3 | Cldn15 | Dstn | Flrt2 | Grb14 | Hist1h4a |
| Hist1h4b | Jam2 | Mest | Ostn | Polr1d | Rnd3 |
| Hist1h4c | Kalrn | Mmp3 | Paqr3 | Ppargc1a | Rnf125 |
| Hist1h4d | Kcnk10 | Mmp9 | Pbk | Ppp1r3c | Rras2 |
| Hist1h4f | Kctd5 | Mmp11 | Pcdh7 | Prc1 | Rrm1 |
| Hist1h4m; Hist1h4n | Kif11 | Mmp13 | Pcdh18 | Prelp | Rrm2 |
| Hist1h4n | Kif20a | Mx1 | Pcdhb12 | Prg4 | Runx1t1 |
| Hist2h3b | Kif26b | Myo10 | Pcdhb16 | Prim1 | S1pr3 |
| Hist2h4 | Klf5 | Naaa | Pde1a | Prkg2 | Scara5 |
| Hmgb2 | Klf9 | Nans | Pde1b | Prmt3 | Scarb2 |
| Hmmr | Klra4; Klra15; Klra1; Klra22; Klra18 | Ncaph | Pde2a | Psmb8 | Scrn1 |
| Hp | Klra5 | Nceh1 | Pde4b | Psmb10 | Sdc3 |
| Hsd11b1 | Kpna2 | Ndc80 | Pdgfrl | Ptgs2 | Sema3a |
| Hsd17b7 | Lbp | Ndufa13; Yjefn3 | Pdpn | Ptn | Serpina3c |
| Id3 | Ldlrad3 | Nfia | Peli2 | Pydc3 | Serpina3m; Serpina3k |
| Ifi30 | Lgals3bp | Nfkbia | Phldb2 | Pyhin1 | Serpinb2 |
| Ifit2 | Lgals4; Lgals6 | Ngf | Pik3ip1 | Qsox1 | Serpinb9b |
| Ifrd1 | Lgals9 | Npr2 | Pitpnc1 | Rab3c | Serpine1 |
| Igf1 | Lilrb4a | Nr4a1 | Pla1a | Rab7b | Serpinf1 |
| Iglon5 | Lpl | Nrg1 | Pla2g15 | Racgap1 | Sfrp2 |
| Iigp1 | Lrig3 | Nuf2 | Plat | Rad51 | Sgk1 |
| Il1rl1 | Lrp5 | Nup35 | Plau | Rap2a | Shcbp1 |
| Il1rn | Lrp6 | Oas1a | Plekhg5 | Rarres2 | Slc1a5 |
| Il15ra | Ltbp1 | Oas1g | Plin1 | Rbp1 | Slc6a6 |
| Il18rap | Manba | Oas2 | Plin4 | Reep6 | Slc7a5 |
| Insig1 | Matn2 | Odc1 | Plk4 | Rgl1 | Slc7a11 |
| Irf1 | Mcm5 | Ogn | Plod1 | Rgs2 | Slc9a6 |
| Isoc1 | Mcm6 | Oip5 | Pltp | Rgs16 | Slc18b1 |
| Itgbl1 | Megf10 | Olfml3 | Plxna1 | Rhou | Slc22a15 |
| Ivl | Mertk | Orm3 | Pola1 | Rnase2a | Slc25a42 |
| Slc27a1 | Sorbs1 | Stom | Timm10 | Tpm2 | Vnn3 |
| Slc29a1 | Sorbs2 | Suv39h2 | Timm17b | Tpm4 | Zbtb16 |
| Slc38a4 | Sort1 | Sycp2 | Timp4 | Tspan11 | Zfpm2 |
| Slc41a3 | Specc1 | Tap1 | Tlr4 | Tspan14 | Zmat3 |
| Slfn8 | Spry3 | Tcf19 | Tm4sf1 | Ttc39c | Zyx |
| Slit2 | Spsb1 | Tcn2 | Tmsb4x | Ttk |  |
| Smim3 | Ssc5d | Thbs1 | Tnc | Tubb6 |  |
| Snai2 | Ssh2 | Thbs2 | Tnfaip6 | Twist2 |  |
| Socs2 | St3gal1 | Tigd2 | Tnn | Ube2c |  |
| Sod3 | Stambpl1 | Timeless | Top2a | Ugcg |  |

**Table S3.** The genes (62 genes) among 403 DEGs (Supplemental Table S2) that are common between the mifepristone-differentiated adipocytes and any of the three epididymal adipose tissues

| Aplp2 | H2-L; H2-D1 | Ldlrad3 | Prg4 | Timm17b |
| --- | --- | --- | --- | --- |
| Cdc45 | Hist1h2af | Lgals4; Lgals6 | Qsox1 | Tm4sf1 |
| Cenpm | Hist1h2ag | Lpl | Rap2a | Tnn |
| Ctsd; Ifitm10 | Hist1h2ah | Manba | Rbp1 | Zfpm2 |
| Dhfr | Hist1h2al | Mcm5 | Rhou | Zmat3 |
| Dhrs7 | Hist1h2ao; Hist1h2ap; Hist1h2ai; Hist1h2ah | Ngf | Rrm1 | Zyx |
| Dstn | Hist1h2ap | Nr4a1 | Scrn1 |  |
| Enpp4 | Hist1h3c | Oip5 | Slc25a42 |  |
| Epb41l4a | Hist1h3g | Peli2 | Slc41a3 |  |
| Epha4 | Hist1h3i | Plat | Smim3 |  |
| Esco2 | Hmgb2 | Plekhg5 | Ssh2 |  |
| Fbxo6 | Irf1 | Plk4 | Suv39h2 |  |
| Gclc | Kalrn | Pola1 | Thbs1 |  |
| Gprc5b | Kcnk10 | Prc1 | Timm10 |  |

adiponectin (A6354; Sigma), 30kDa

adipocyte fatty acid binding protein-4 (Fabp4) (cat.3544; Cell Signaling Technology), 15kDa

PPARγ (cat.2430; Cell Signaling Technology), 53, 57kDa

Rac1 (cat.BD610650; BD Biosciences, Franklin Lakes, NJ, USA), 21kDa

cyclophilin B (cat.ab16045; Abcam, Cambridge, MA, USA), 21kDa

Size Marker: Prestained Protein Marker, Broad Range (cat.#7720; Cell Signaling Technology)
